# Multiplexed Proteome Dynamics Profiling Reveals Mechanisms Controlling Protein Homeostasis

**DOI:** 10.1016/j.cell.2018.02.030

**Published:** 2018-03-22

**Authors:** Mikhail M. Savitski, Nico Zinn, Maria Faelth-Savitski, Daniel Poeckel, Stephan Gade, Isabelle Becher, Marcel Muelbaier, Anne J. Wagner, Katrin Strohmer, Thilo Werner, Stephanie Melchert, Massimo Petretich, Anna Rutkowska, Johanna Vappiani, Holger Franken, Michael Steidel, Gavain M. Sweetman, Omer Gilan, Enid Y.N. Lam, Mark A. Dawson, Rab K. Prinjha, Paola Grandi, Giovanna Bergamini, Marcus Bantscheff

**Affiliations:** 1 Cellzome GmbH, GlaxoSmithKline, Meyerhofstrasse 1, 69117 Heidelberg, Germany; 2 Genome Biology Unit, European Molecular Biology Laboratory, 69117 Heidelberg, Germany; 3 Cancer Research Division, Peter MacCallum Cancer Centre, East Melbourne, VIC 3002, Australia; 4 Epinova DPU, Immuno-Inflammation Centre of Excellence for Drug Discovery, GlaxoSmithKline, Medicines Research Centre, Gunnels Wood Road, Stevenage SG1 2NY, UK

**Keywords:** proteostasis, mass spectrometry, degradation, protein turnover, HSP90, PROTAC, mechanism-of-action, JQ1, estrogen receptor

## Abstract

Protein degradation plays important roles in biological processes and is tightly regulated. Further, targeted proteolysis is an emerging research tool and therapeutic strategy. However, proteome-wide technologies to investigate the causes and consequences of protein degradation in biological systems are lacking. We developed “multiplexed proteome dynamics profiling” (mPDP), a mass-spectrometry-based approach combining dynamic-SILAC labeling with isobaric mass tagging for multiplexed analysis of protein degradation and synthesis. In three proof-of-concept studies, we uncover different responses induced by the bromodomain inhibitor JQ1 versus a JQ1 proteolysis targeting chimera; we elucidate distinct modes of action of estrogen receptor modulators; and we comprehensively classify HSP90 clients based on their requirement for HSP90 constitutively or during synthesis, demonstrating that constitutive HSP90 clients have lower thermal stability than non-clients, have higher affinity for the chaperone, vary between cell types, and change upon external stimuli. These findings highlight the potential of mPDP to identify dynamically controlled degradation mechanisms in cellular systems.

## Highlights

• Multiplexed proteome dynamics profiling, mPDP, measures changes in proteostasis
• JQ1-PROTAC degrades a key mRNA export factor and blocks protein synthesis
• Raloxifene induces TMEM97 degradation dysregulating cholesterol homeostasis
• Characterization of proteins dependent on HSP90 constitutively or during synthesis

## Graphical Abstract

**Figure:**
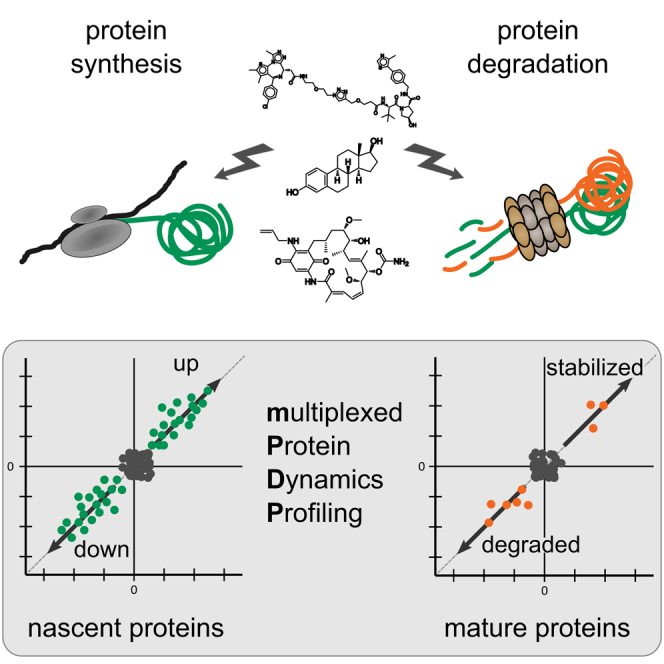

## Teaser

Tracking both protein synthesis and degradation across thousands of proteins yields insights into functional regulation by protein degradation.

## Introduction

Protein homeostasis integrates the balanced control of protein synthesis and degradation with the regulation of mRNA metabolism. External stimuli and disease can modulate any of these processes and thus alter the proteome. Similarly to protein synthesis, the regulation of protein degradation is complex involving hundreds of proteins such as E3 ligases, deubiquitinases, and proteases to orchestrate proteasomal, lysosomal, and other degradation pathways (Goldberg, 2003, Labbadia and Morimoto, 2015). Although it is known that many transcription factors and other regulatory proteins are characterized by fast turnover and rapid proteasomal degradation (e.g., ESR1, HIF1A), our understanding of how protein degradation controls dynamic biological processes is still emerging.

Small-molecule based strategies to target specific proteins for degradation have been developed recently (Lai and Crews, 2017) and offer exciting opportunities not only for chemical biology investigations but also for therapeutic intervention. Selective protein degradation has the potential to complement current inhibitor strategies by enabling the ablation of scaffolding activities as well as low and infrequent dosing when targeting proteins with slow synthesis rates (Deshaies, 2015). Protein degradation can be induced by inhibiting protein-protein interactions with chaperones (Ahsan et al., 2013), destabilizing protein structure with inhibitors (Wu et al., 2005), or directing proteins to the proteasome with “proteolysis targeting chimeras” (PROTACs) (Lai and Crews, 2017). PROTACs consist of a small ligand with high affinity to the target protein that is connected to a second ligand that recruits an E3 ubiquitin ligase. Upon formation of a ternary complex the targeted protein is ubiquitylated and degraded. Conventional proteomics analyses detect changes in protein abundances for a large fraction of the proteome but provide little differentiation between targets and downstream regulatory effects of protein knockdown (Bondeson et al., 2015).

In recent years methodologies have become available to measure the activity of degradation mechanisms by analyzing protein turnover (Pratt et al., 2002), synthesis, and degradation rates (Boisvert et al., 2012, Jovanovic et al., 2015) with pulsed (Schwanhäusser et al., 2009) or dynamic stable isotope labeling with amino acids in cell culture (SILAC) (Doherty et al., 2009, Schwanhäusser et al., 2011) also in combination with isobaric mass tagging (Welle et al., 2016). Notwithstanding these technological advances, the discovery of regulated degradation mechanisms in biological processes and upon pharmacological intervention is still hampered by the lack of a sensitive global profiling approach to distinguish dynamic changes in protein synthesis and degradation across a range of conditions at high throughput.

Here, we present multiplexed proteome dynamics profiling (mPDP). This quantitative liquid chromatography-tandem mass spectrometry (LC-MS/MS)-based method combines dynamic SILAC labeling with isobaric mass tagging to enable the sensitive and comprehensive simultaneous analysis of changes in protein degradation and synthesis in a single mass spectrometric experiment of biological replicates for multiple treatment conditions. To demonstrate the broad applicability of mPDP for uncovering dynamic regulation of protein degradation mechanisms, we applied this strategy to investigate proteome effects by PROTAC-mediated degradation of the BET transcription factor family, ligand-induced degradation of the estrogen receptor, and inhibition of the chaperone heat shock protein 90 (HSP90).

## Results

### Measuring the Impact of Cellular Perturbations on Proteome Homeostasis

**Figure 1:**
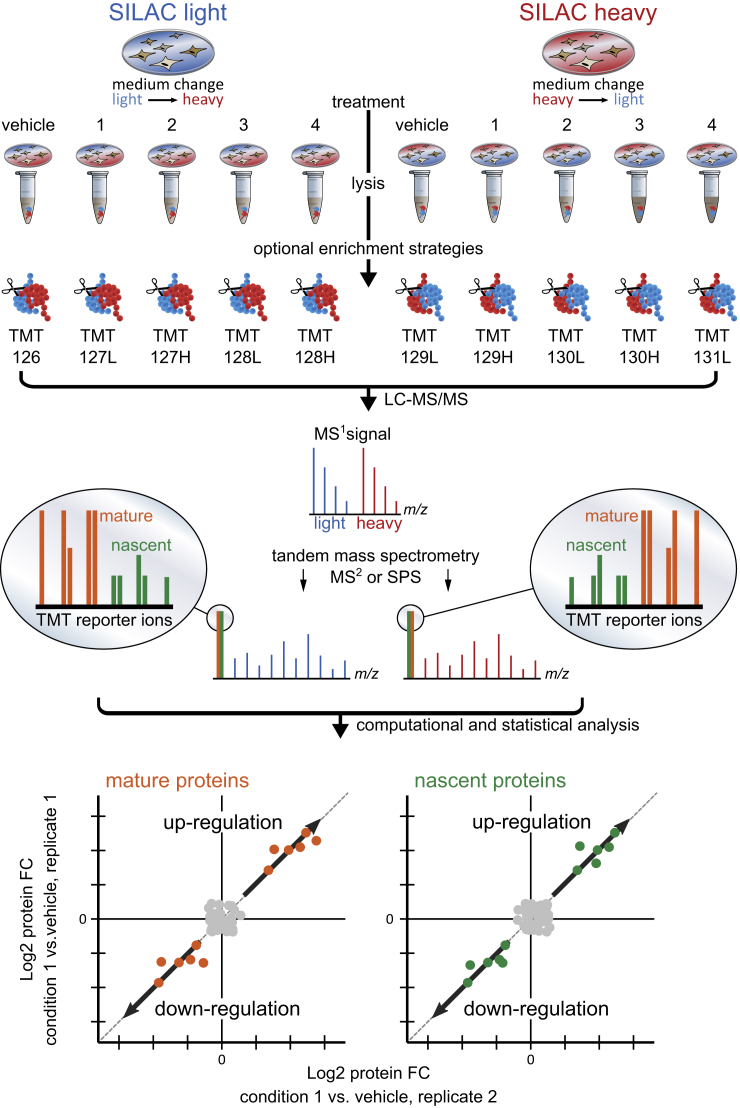
Experimental Scheme of mPDP

**Figure S1:**
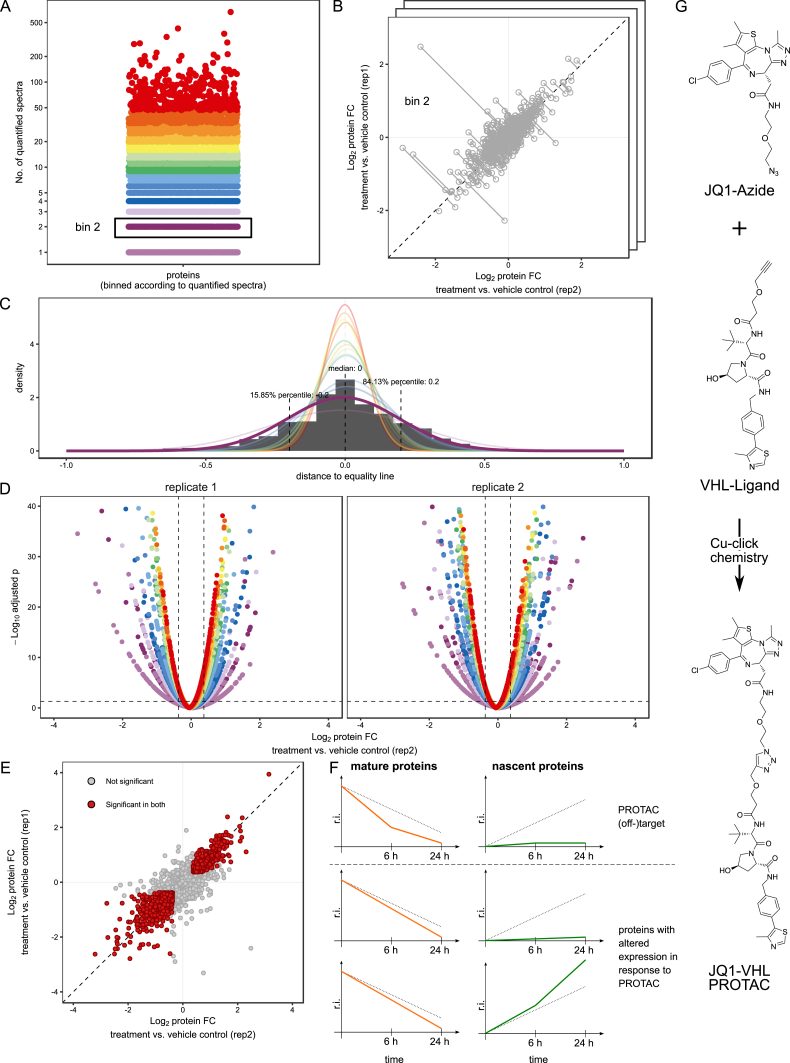
Statistical Analysis of mPDP Experiments, Related to Figure 1 (A) Step 1: Proteins were divided into bins according to the number of quantified spectrum sequence matches (color coding according to bins). Each bin consisted of at least 300 proteins. This data quality–dependent binning strategy is analogous to the procedure described previously (see the STAR Methods). (B) Step 2: The standard deviation per bin was calculated using robust estimation (using the 15.87, 50, and 84.13 percentiles) from the distribution of differences between the log_2_-transformed fold changes (FC) of the same proteins from two biological replicates divided by the square root of two: $\frac{\left({\text{log}}_{2}\left({\text{FC}}_{\text{rep}1}\right)-{\text{log}}_{2}\left({\text{FC}}_{\text{rep}2}\right)\right)}{\sqrt{2}}.$ This metric can be visualized as the distance to the unity line when plotting a scatterplot of protein log_2_ fold changes from two replicates. The metric measures the shortest distance to the unity line as illustrated in the figure. By using the standard deviation from the differences in log_2_ fold changes of proteins between two biological replicates the reproducibility between the replicates is taken into account in the statistical test. This enables to assess biologically relevant regulation upon perturbation without having to adhere to the assumption that the majority of measured proteins are not regulated. (C) Density plots of the distance to equality line for each bin (color code according to [A]). Step 3: For each protein log_2_ fold change a p value was calculated using a Z-test with a robust estimation of the standard deviation calculated in step 2. Step 2 and step3 were performed on each bin separately. (D) Volcano plots displaying the protein fold change (FC) versus -log_10_ adjusted p value. Color code as in (A). Step 4: After step 2 and 3 have been completed for all bins, adjustment for multiple hypothesis testing was performed on the full data set by using Benjamini-Hochberg correction. (E) Scatterplots showing protein log_2_ fold changes (FC). Red closed circles indicate statistically significant regulation (p < 0.05) and the dashed diagonal indicates the equality line. Step 5: Proteins were considered as significantly regulated if the following three conditions were fulfilled: (1) the adjusted p value was ≤ 0.05 in both replicates and (2) absolute protein log2 FC was greater than 0.37 in both replicates and (3) the change was in the same direction in both replicates. (F) Schematic representation of the expected behavior of PROTAC direct targets on mature and newly synthesized proteins over time (upper panel). Expected behavior of protein indirectly affected, e.g., due to downstream effects (lower panel). Changes in protein levels are displayed as relative intensity (r.i.). (G) Modular design of JQ1-VHL PROTAC: JQ1-Azide Cu-click chemistry reaction with VHL-Ligand to generate JQ1-VHL PROTAC.

To elucidate how cellular perturbations affect protein synthesis and degradation, we developed mPDP (Figure 1). This approach enables the sensitive and comprehensive relative quantification of cellular mature and nascent protein pools across a range of different treatment conditions in biological duplicates and in a single mass spectrometric experiment by combining dynamic SILAC with chemical labeling using neutron-encoded tandem mass tags (TMT) (McAlister et al., 2012, Welle et al., 2016, Werner et al., 2012). In one replicate, cells are grown in light SILAC medium containing arginine ^12^C_6_^14^N_4_ and lysine ^12^C_6_^14^N_2_ and switched to the heavy SILAC medium containing stable isotope-enriched amino acids arginine ^13^C_6_^15^N_4_ and lysine ^13^C_6_^15^N_2_ followed by different compound treatments. A biological replicate experiment is performed in parallel with cells grown in SILAC heavy medium, where the same treatments are applied after medium exchange to SILAC light medium. After cell lysis, optional affinity enrichment, e.g., with kinobeads (Bantscheff et al., 2007) is performed, followed by trypsinolysis and chemical labeling with TMT reagents. Subsequently, all samples are combined and subjected to LC-MS/MS. This sample treatment and mixing scheme leads to very similar signal intensities of SILAC pairs, and to a high percentage of peptides fragmented in both states and hence few missing values in biological duplicates. Tandem mass spectra of SILAC light and heavy-encoded peptide ion signals each contain reporter ions of pre-existing peptides (mature) of the first and newly synthesized peptides (nascent) of the second replicate experiment or vice versa. For mature proteins, upregulation indicates slower degradation in the treated sample while downregulation suggests faster degradation. For nascent proteins, upregulation indicates increased protein production due to transcriptional or posttranscriptional regulation. If upregulation is also observed for the mature protein it can also indicate slower degradation. Likewise, downregulation of nascent proteins either indicates reduced protein production or enhanced degradation if the mature protein form is also downregulated. Reproducibility of quantitative protein measurements between biological replicates was used to assess variance and to identify significantly regulated proteins including cases where larger portions of the proteome were affected (Figures S1A–S1E). We applied this strategy to investigate how three different small molecules that induce protein degradation affect the proteome: (1) a PROTAC, (2) estrogen receptor ligands, and (3) an HSP90 inhibitor.

### PROTACs Display On- and Off-Target Effects

First, we applied mPDP to study proteome effects of the bromodomain and extra-terminal domain (BET) family inhibitor JQ1 and a JQ1-VHL PROTAC. The BET proteins BRD2, 3, and 4 and BRDT regulate gene expression by recognizing acetylated histones in open chromatin conformations. Hence, BET inhibitors and degraders alter protein levels by gene repression and potentially by indirectly affecting degradation (Dawson et al., 2011, Filippakopoulos et al., 2010). We rationalized that mPDP would enable distinguishing PROTAC-induced degradation events from downstream transcriptional or translational regulation as altered degradation rates are detected in the mature and nascent protein pools, whereas downstream effects leading to altered synthesis rates should be exclusively detected among the nascent proteins synthesized during treatment (Figure S1F).

**Figure 2:**
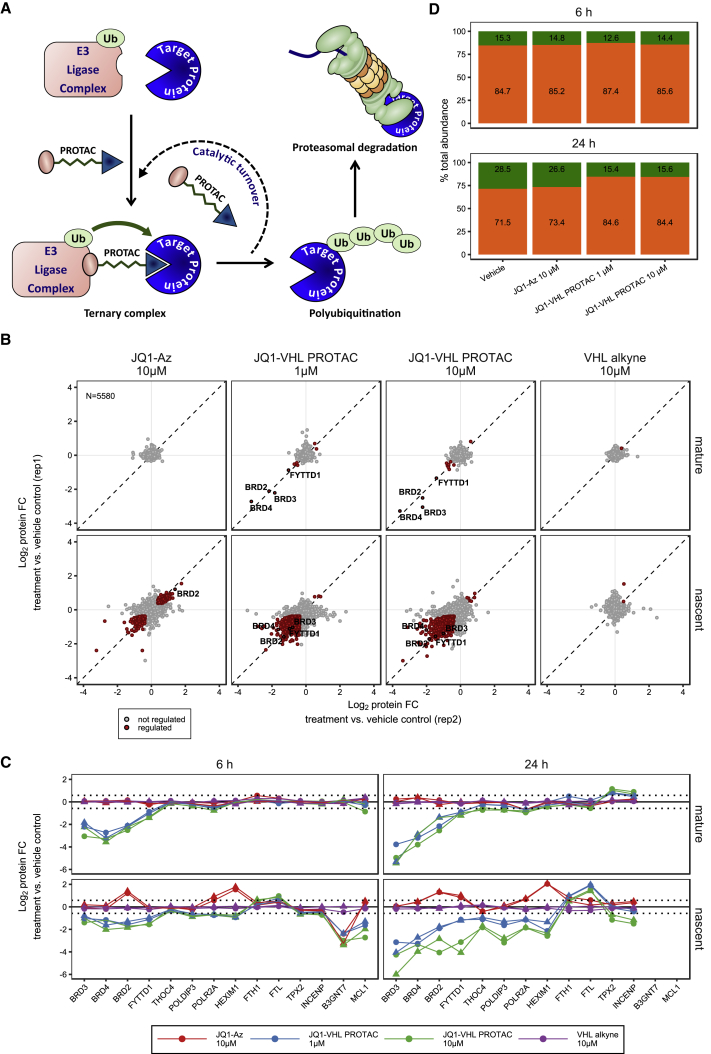
Mechanism of Action of a JQ1-VHL PROTAC (A) Schematic representation of the mechanism of action of a PROTAC. (B) Scatterplots of protein fold changes (FC) observed in mature (upper panel) and nascent (lower panel) forms of proteins in THP-1 cells treated for 6 hr with the BET inhibitor JQ1-Az (10 μM), the JQ1-VHL PROTAC (1 and 10 μM), or VHL alkyne (10 μM) relative to vehicle control in two biological replicate experiments. Red closed circles indicate statistically significant regulation (p < 0.05), and BRD2, 3, and 4 and FYTTD1 are labeled exemplarily. The dashed diagonal indicates the equality line. Upper panels indicate regulation of proteins that were already present before compounds were added (mature proteins), and lower panels indicate proteins synthesized after compounds were added (nascent proteins). N indicates the number of proteins robustly quantified in both replicates. (C) Line charts with markers of protein fold changes observed in mature (upper panel) and nascent (lower panel) forms of selected proteins after 6- (left) and 24-hr (right) treatment with JQ1-Az (10 μM, red), JQ1-VHL PROTAC (1 μM, blue; 10 μM, green), and VHL alkyne (10 μM, purple) relative to vehicle control. Triangles and circles distinguish data from biological replicates 1 and 2. Proteins where fold changes measured for both replicates are outside the dotted lines are significantly regulated (p < 0.05). (D) Bar chart indicating relative contributions of nascent (green) and mature (orange) proteins to proteome composition after 6- or 24-hr incubation with vehicle, JQ1-Az (10 μM), and JQ1-VHL PROTAC (1 and 10 μM). Percentages of mature and nascent proteins are estimated from the summed-up reporter ion abundances.

**Figure S2:**
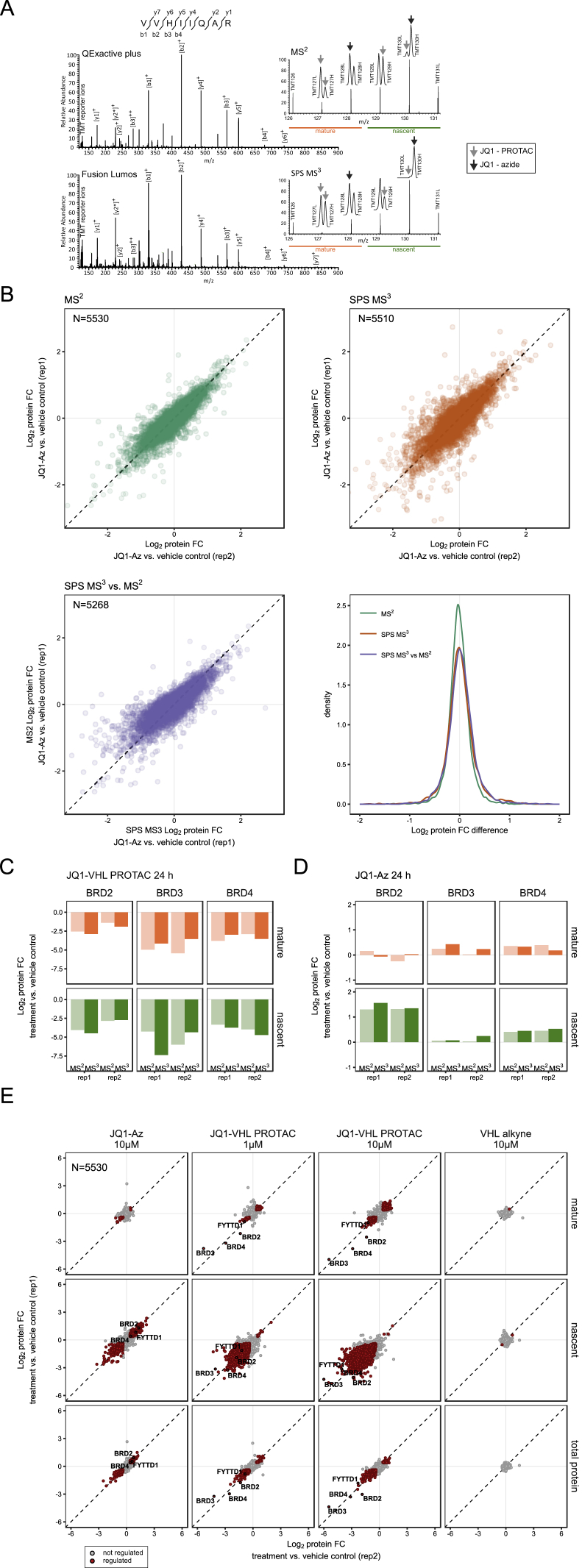
Assessment of Reproducibility of Biological Replicates and Technical Validation of the Quantification, Related to Figure 2 (A–E) Samples were analyzed on a Q-executive (using MS^2^ for reporter ion quantification) as well as on a Fusion Lumos (using SPS MS^3^ for reporter ion quantification) mass spectrometer to assess the reproducibility of biological replicates and as a technical validation of the quantification. (A) Annotated MS^2^ and MS^3^ spectra for the BRD2 peptide VVHIIQAR. (B) The first two scatterplots compare protein fold changes (FC) between two biological replicates using MS^2^ and MS^3^ based quantification respectively, the third scatterplot compares fold changes calculated using either MS^2^ or MS^3^ based quantification performed on aliquots from the same samples. The density plots show the difference of protein fold changes between the biological replicates in the first and second plot and the difference between protein fold changes from MS^2^ and MS^3^ based quantification in the third plot. (C) Protein fold changes (FC) observed in mature (upper panel) and nascent (lower panel) forms of proteins in THP-1 cells treated for 24 h with 10 μM JQ1-VHL PROTAC relative to vehicle, for BRD2, BRD3 and BRD4 using MS^2^ and MS^3^ quantification in two biological replicates. (D) Protein fold changes (FC) observed in mature (upper panel) and nascent (lower panel) forms of proteins in THP-1 cells treated for 24 h with 10 μM JQ1-Az relative to vehicle, for BRD2, BRD3 and BRD4 using MS^2^ and MS^3^ quantification in two biological replicates. (E) Scatterplots showing protein fold changes (FC) observed in mature forms (upper panel), nascent forms (middle panel) and the total proteome level estimated from the summed-up reporter ion abundances over mature and nascent proteins (lower panel) of proteins in THP-1 cells treated for 24 h with JQ1-Az (10 μM), JQ1-VHL PROTAC (1 and 10 μM) or VHL alkyne (10 μM) relative to vehicle treated cells. Red closed circles indicate statistically significant regulation (p < 0.05) and the dashed diagonal indicates the equality line. N indicates the number of proteins robustly quantified in both replicates.

We synthesized a BET bromodomain PROTAC by coupling an azide-containing analog of the BET inhibitor JQ1 to an alkyne-derivatized peptidomimetic of the HIF1Α hydroxyproline-containing sequence motif known to bind to the Von Hippel-Lindau E3 ubiquitin ligase complex (VHL) (Buckley et al., 2012, Wurz et al., 2018) (Figures 2A and S1G). Then we treated THP-1 cells for 6 and 24 hr with JQ1-azide (JQ1-Az), the JQ1-VHL PROTAC (1 and 10 μM), the VHL-binding compound (VHL alkyne), or vehicle and processed the samples with the mPDP workflow (Figure 2B). The samples of the 24-hr time point were measured in parallel using standard tandem mass spectrometry, MS^2^, on a Q-Exactive+ mass spectrometer and using the SPS MS^3^ approach (McAlister et al., 2014) on an Orbitrap Lumos system. Both approaches yielded comparable proteome coverage with the MS^3^ data displaying slightly more pronounced fold changes but lower reproducibility (Figures S2A–S2D); therefore we used MS^2^ for all subsequent experiments.

After 6 hr JQ1-Az treatment, no significant effects were observed for mature proteins but substantial regulation was detected for nascent proteins, such as the N-acetylgalactosamine transferase B3GNT7, BRD2, HEXIM1, and POLR2A in agreement with recent reports (Bhadury et al., 2014, Huang et al., 2016, Lovén et al., 2013). In contrast, the JQ1-VHL PROTAC induced the expected rapid degradation of BET family members BRD2, 3, and 4, as indicated by substantially reduced abundances in the mature and nascent protein pools (Figures 2B and 2C). Thus, these data validate the mPDP approach for differentiating treatment-dependent effects on mature and nascent proteins. Interestingly, forty-two-three domain-containing protein 1 (FYTTD1/UIF), an adaptor of the transcription/export complex TREX (Hautbergue et al., 2009) was also degraded by JQ1-VHL PROTAC followed by depletion of the other TREX complex components THOC4 and POLDIP3 after 24 hr (Figures 2C and S2C).

Nascent protein levels observed after 6 hr treatment with the JQ1-VHL PROTAC largely recapitulated the response observed with JQ1-Az, with exception of the direct PROTAC targets and of a small cluster of proteins including the BCL2-like antiapoptotic protein MCL1. However, upon 24-hr JQ1-VHL PROTAC treatment, accumulation of mature proteins typically upregulated in mitosis, such as the AURKA interactors TPX2, INCENP, and kinesins, suggested cell-cycle arrest in the G2/M transition. Further, enhanced degradation of POLR2A was observed (Figures 2C and S2E). When comparing the relative contribution of newly synthetized proteins to the total protein levels, almost no further increase in nascent proteins was observed after the 6-hr time point, and only ferritins (FTH1, FTL) were upregulated (Figure 2C).

**Figure 3:**
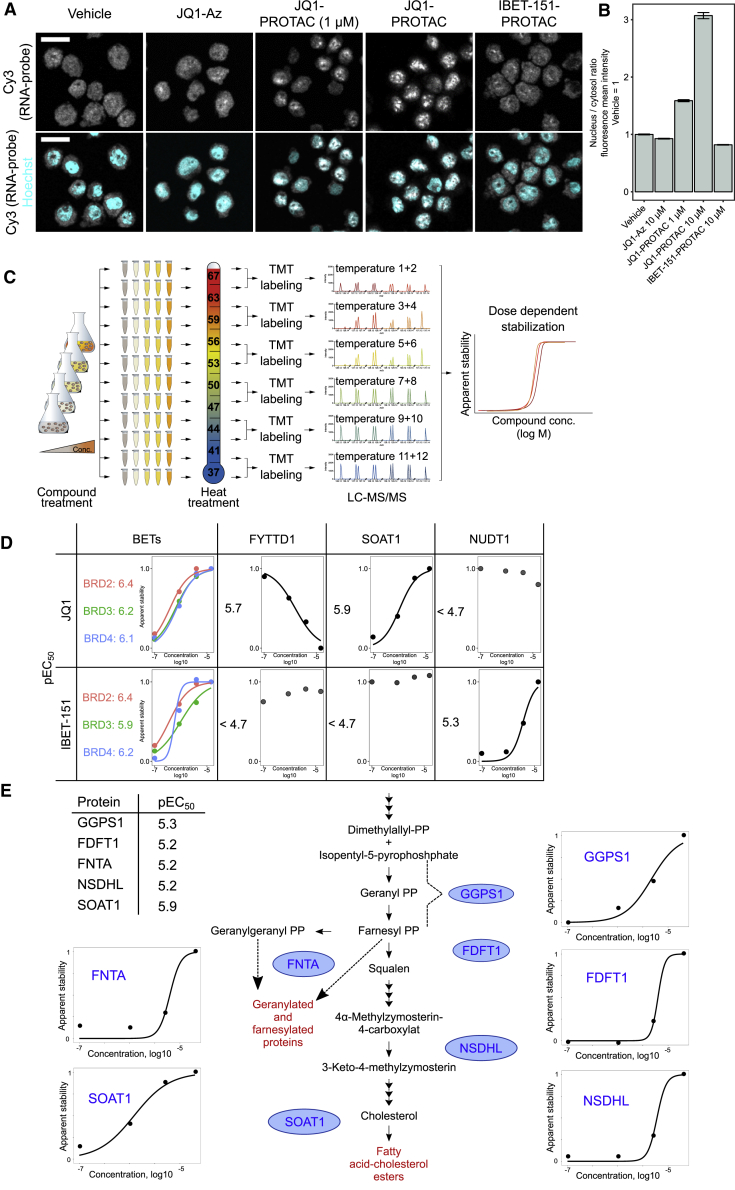
Off-Target Effects of JQ1 and the JQ1-VHL-PROTAC (A) Imaging of nuclear RNA content by fluorescence *in situ* hybridization (FISH) and confocal microscopy. THP-1 cells were treated with vehicle, JQ1-Az (10 μM), JQ1-VHL-PROTAC (at 1 and 10 μM), or I-BET-151-VHL-PROTAC (10 μM) for 6 hr, fixed, and processed for FISH using Cy3-labeled oligo-dT50. Nuclei were stained by Hoechst. Representative fluorescent images recorded after excitation at 514 nm (Cy3, gray, upper panel) are shown. The lower panel displays an overlay of Cy3 staining (gray) and Hoechst staining (cyan). Scale bar, 20 μm. (B) Bar chart displaying the ratio of mean fluorescence intensity of the FISH probe (Cy3 channel) between nucleus (defined by Hoechst staining) and cytosol (cell borders as defined by WGA staining) was calculated for single cells (706–1,215 cells per condition). Mean fluorescence of treated samples is normalized to control vehicle. SEM is shown. The experiment was repeated three times (Figures S3A and S3B). (C) Scheme of 2D thermal proteome profiling (2D-TPP) experiments. (D) 2D-TPP results for JQ1 and I-BET151. Sigmoidal curves show dose-dependent changes in thermal stability for selected proteins. pEC_50_ is defined as – log_10_(EC_50_). (E) Dose-dependent effects of cellular JQ1 treatment on the thermal stability of five proteins involved in cholesterol biosynthesis revealed by 2D-TPP. The table shows pEC_50_s for dose-dependent stabilization; the pathway is displayed in the center, and enzymes are marked in blue. Curves depict dose-dependent stabilization in JQ1-treated cells for indicated enzymes.

**Figure S3:**
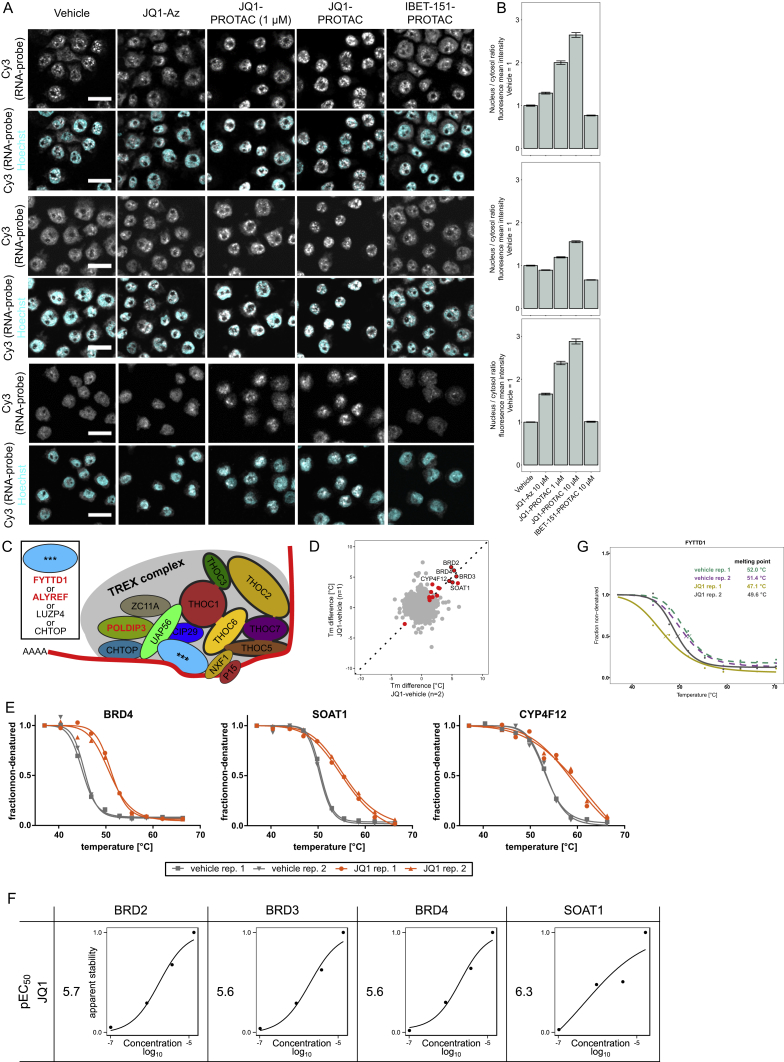
Off-Target Effects of JQ1 and the JQ1-VHL-PROTAC, Related to Figure 3 (A) Fluorescence *in situ* hybridization (FISH) of polyA+RNA detected by a Cy3-labeled oligo-dT50 probe. THP-1 cells were treated with either vehicle, JQ1-Az (10μM), JQ1-VHL-PROTAC (at 1 or 10 μM) or I-BET-151-VHL-PROTAC (10 μM) for 6 hr (3 independent experiments are displayed). Cellular localization of polyA+RNA was visualized with confocal microscopy. Representative fluorescent images recorded after excitation at 514 nm (Cy3, gray) are shown. Lower panel displays an overlay of Cy3 staining (gray) and Hoechst nuclear staining (cyan) Scale bar, 20 μm. (B) Ratio of mean fluorescence intensity of FISH probe (Cy3 channel) between nucleus (defined by Hoechst staining) and cytosol (cell borders were defined by WGA staining, not shown) was calculated for single cells using the CellProfiler software (520-790 cells for experiment 1, 546-791 cells for experiment 2 and 278-578 cells for experiment 3 were quantified per condition) from experiment displayed in (A). SEM is shown. (C) Schematic representation of the TREX complex components. Protein names in red text are found significantly regulated compared to vehicle control upon treatment with JQ1-VHL PROTAC. (D) Thermal proteome profiling (TPP) experiment in HepG2 cells after incubation with 15 μM JQ1 (Tm difference in degree Celsius of JQ1 versus vehicle). Proteins with strongest stabilization in two independent replicates are highlighted. Red color indicates significant changes (E) Thermal stabilization of BRD4, SOAT1 and CYP4F12 induced by 15 μM JQ1 in HepG2 cells displayed as normalized non-denatured protein fraction. (F) 2D-thermal proteome profiling of JQ1 in THP1 cell lysate. Dose-dependent stabilization of BRD2, 3, 4 and SOAT1 are displayed (normalized apparent stability and pEC_50_ are reported) (G) Thermal shift assay of recombinant FYTTD1 with JQ1.

To investigate whether degradation of FYTTD1 and concomitant impairment of the mRNA nuclear export mediated by the TREX complex, contributed to the observed protein synthesis arrest, we measured nuclear RNA content by fluorescence *in situ* hybridization (FISH) and confocal microscopy (Figures 3A, 3B, and S3A–S3C). Substantial RNA accumulation was observed in nuclei of THP-1 cells treated with JQ1-VHL PROTAC, but not with the inhibitor JQ1-Az or a PROTAC based on the alternative BET inhibitor I-BET151 (Dawson et al., 2011). Two-dimensional thermal proteome profiling (2D-TPP) experiments (Becher et al., 2016) with JQ1 and I-BET151 (Figure 3C) were performed to further investigate whether FYTTD1 is a direct target of JQ1 and whether additional JQ1 off-targets could contribute to the observed effects on mRNA export. The BET proteins were stabilized by both compounds, with submicromolar EC_50_s, confirming intracellular target engagement in THP-1 cells (Figure 3D). JQ1 further caused dose-dependent destabilization of FYTTD1 and stabilized SOAT1 and several members of the sterol biosynthesis pathway (Figures 3D and 3E), with approximately 1 μM EC_50_s. In contrast, I-BET151 had a distinct target profile, stabilizing NUDT1 but not affecting FYTTD1 or SOAT1 (Figure 3D). Direct binding of JQ1 to SOAT1 was confirmed in TPP experiments performed in THP-1 cell extracts and in HEPG2 cells (Figures S3D–S3F). Other enzymes in the cholesterol synthesis pathway were not stabilized in cell extracts, suggesting that their stabilization in cell-based experiments is an indirect consequence of SOAT1 binding. Thermal shift assays with recombinantly expressed FYTTD1 confirmed destabilization by JQ1 binding (Figure S3G).

In summary, we identified the TREX complex adaptor FYTTD1 and the transmembrane endoplasmic reticulum (ER) protein SOAT1 as JQ1 off-targets. The JQ1-VHL PROTAC caused ablation of FYTTD1 and led to full arrest of protein synthesis. The results indicate that off-target activities substantially contribute to molecular and phenotypic responses observed upon JQ1 and JQ1-PROTAC treatment.

### Ligand-Induced Degradation of the Estrogen Receptor

**Figure 4:**
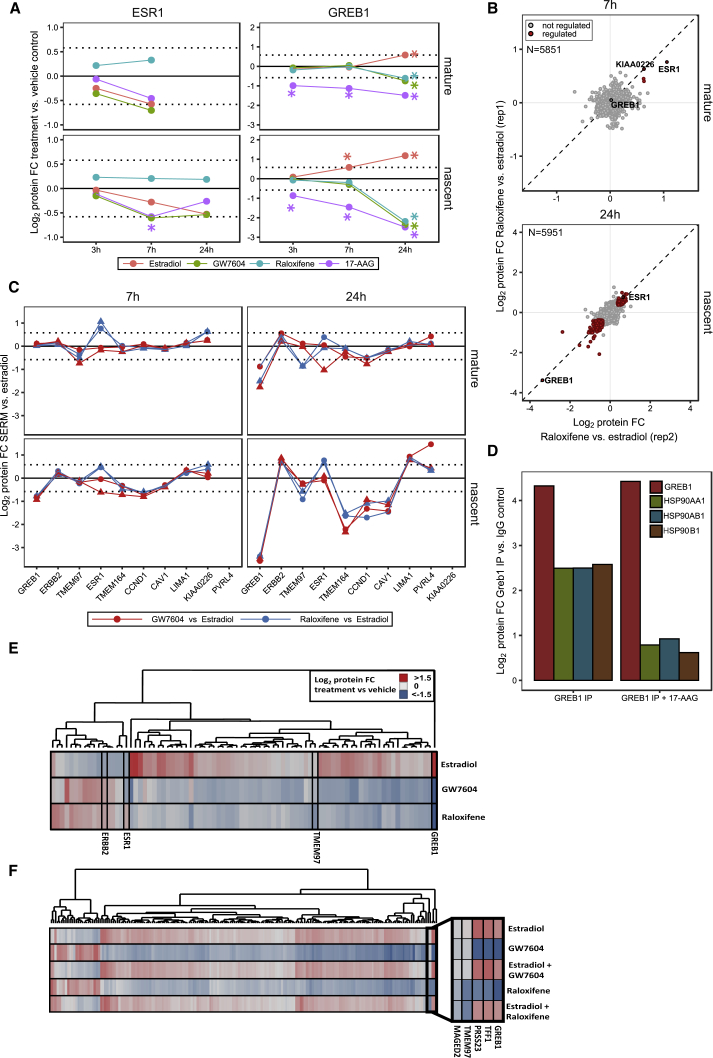
Effects of Estrogen and SERMs on Proteostasis (A) Line charts of protein fold changes (FC) for time-dependent regulation of mature (upper panels) and nascent (lower panels) levels of ESR1 and Greb1 upon treatment of MCF-7 cells with vehicle, estradiol, GW7604, raloxifene, or 17-AAG. An asterisk indicates significant regulation. p < 0.05. (B) Scatterplots of protein fold changes for mature (upper panel) and nascent (lower panel) proteins in MCF-7 cells treated for 7 (upper panel) or 24 hr (lower panel) with raloxifene relative to estradiol treated cells. Red closed circles mark statistically significant regulation (p < 0.05), and the dashed diagonal is the equality line. N is the number of proteins robustly quantified in both replicates. (C) Line charts with markers of protein fold changes for selected (upper panel) and nascent (lower panel) proteins after 7 (left) and 24 hr (right) treatment with GW7604 (red line) and raloxifene (blue) relative to estradiol. Triangles and circles distinguish data from biological replicates 1 and 2. Proteins where fold changes for both replicates are outside the dotted lines are significantly regulated (p < 0.05). (D) Bar chart representation of fold changes determined by mass spectrometry for GREB1, HSP90AA1, HSP90AB1, and HSP90B1 in GREB1 co-immunoprecipitation experiments from cell lysate incubated with vehicle (left) or 10 μM 17-AAG (right) relative to isotype matched immunoglobulin G (IgG) control samples. (E) Heatmap and hierarchical cluster analysis of significantly (p < 0.05) regulated nascent proteins after 24-hr incubation with estradiol, GW7604, or raloxifene. The color scheme depicts regulation relative to vehicle control samples. (F) Heatmap and hierarchical cluster analysis of proteins significantly (p < 0.05) regulated in abundance upon 24-hr incubation with estradiol, GW7604, estradiol + GW7604, raloxifene, or estradiol + raloxifene. The zoomed-in area shows two distinct sub-clusters. The same color scheme used in (E) was used here. See also Table S1 and Table S2.

**Figure S4:**
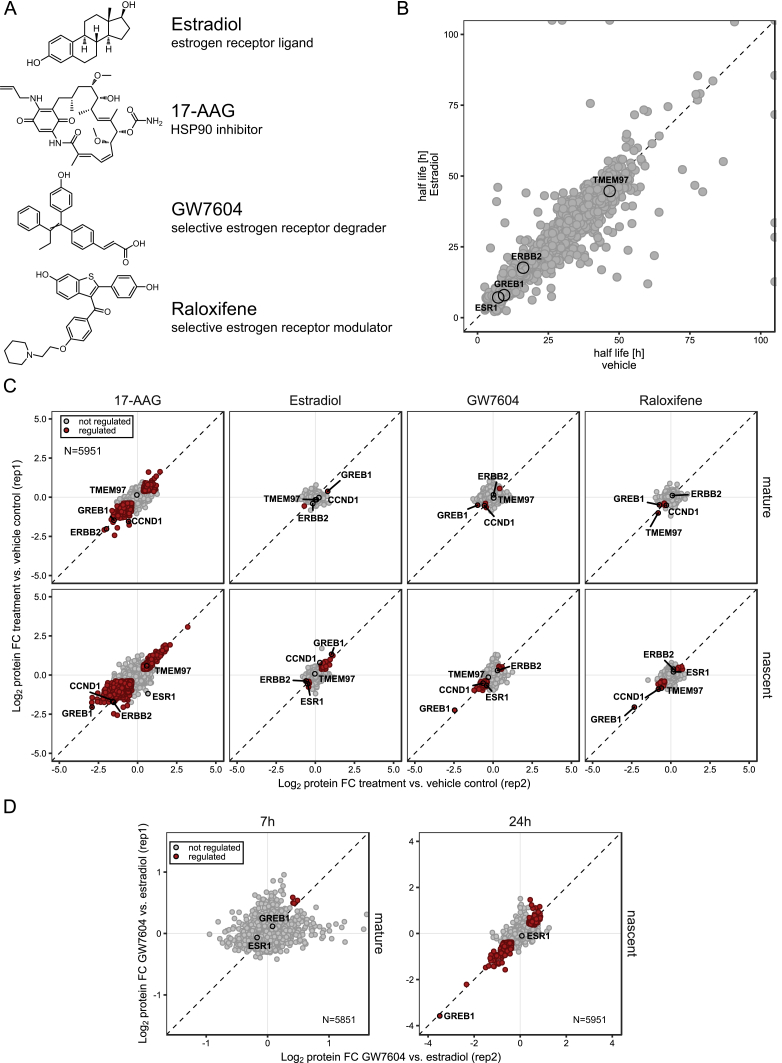
Effects of Estrogen and SERMs on Proteostasis, Related to Figure 4 (A) Compounds structure of estradiol, 17-AAG, GW7604, raloxifene used in experiments reported in Figures 4 and S4. (B) Scatterplot of protein half-lives (displayed in h) determined by dynamic SILAC in MCF7 cells treated with vehicle or estradiol. The dashed diagonal indicates the equality line. (C) Scatterplots showing protein fold changes (FC) observed in mature (upper panel) and nascent (lower panel) forms of proteins in MCF-7 cells treated for 24 h with 17-AAG, estradiol, GW7604 or raloxifene relative to vehicle treated cells. Red closed circles indicate statistically significant regulation (p < 0.05) and the dashed diagonal indicates the equality line. N indicates the number of proteins robustly quantified in both replicates. (D) Scatterplots showing protein fold changes (FC) observed in mature (left panel) and nascent (right panel) forms of proteins in MCF-7 cells treated for 7 h (left panel) or 24 h (right panel) with GW7604 relative to estradiol treated cells. Red closed circles indicate statistically significant regulation (p < 0.05) and the dashed diagonal indicates the equality line. N indicates the number of proteins robustly quantified in both replicates.

Next, we investigated how the estrogen receptor 1 (ESR1) agonist estradiol and the selective estrogen receptor modulators (SERMs) raloxifene and GW7604 affect proteostasis in MCF-7 breast cancer cells (Figure S4A). Estradiol treatment selectively reduced mature and nascent ESR1 levels, suggesting enhanced degradation, in line with previous reports (Alarid et al., 1999, Nawaz et al., 1999) (Figure 4A). ESR1 degradation was substantially slower in raloxifene-treated cells than in estradiol-treated cells but did not change significantly compared to vehicle treated cells (Figures 4A–4C). The estradiol-dependent effects on degradation of the HSP90 client ESR1 could only be detected with mPDP but not in dynamic SILAC experiments (Figure S4B) and were comparable to those observed with the HSP90 inhibitor 17-AAG (Fliss et al., 2000) (Figure 4A). Treatment with GW7604 led to enhanced ESR1 degradation (Figure 4A) consistent with the drug reducing receptor stability due to structural distortions (Wu et al., 2005).

We further found that levels of the nascent estrogen-responsive protein GREB1 increased following treatment with estradiol and decreased with SERMs (Figures 4C and S4C) in agreement with the reported regulation of GREB1 by ESR1 activity (Ghosh et al., 2000). All tested treatments altered the levels of mature GREB1 after 24 hr, whereas mature ESR1 levels changed already after 7 hr. GREB1, like ESR1, was degraded in the presence of 17-AAG and HSP90 proteins co-immunoprecipitated with GREB1 in a 17-AAG-dependent manner (Figure 4D; Table S1). These results indicate GREB1 as HSP90 client and suggest that its degradation can be modulated by ESR1 activity. In addition, the regulation of proteins in the nascent protein pools, e.g., CAV1, CCND1, TMEM164, and ERBB2 was consistent with ESR1-responsive gene regulation (Figure 4C; Table S2), and both SERMs induced similar changes (e.g., nascent PVRL4 and LIMA1), mostly diametrical to estradiol (Figures 4C, 4E, and S4D). Interestingly, raloxifene elicited additional effects on proteins that were not affected by estradiol or GW7604, suggesting activities unrelated to ESR1 modulation. These included stabilization of the VPS34 complex component Rubicon (KIA0226) and enhanced degradation of the transmembrane protein TMEM97, a regulator of cellular cholesterol homeostasis (Bartz et al., 2009). TMEM97 regulation as an off-target activity of raloxifene was confirmed by comparing proteome levels in cells either treated with estradiol or SERMS or with estradiol and SERMs. Co-treatment with estradiol reverted all raloxifene-induced expression changes to levels observed with estradiol alone, except for MAGED2 and TMEM97 (Figure 4F).

Taken together, our results demonstrate the ability of mPDP to detect modest changes in protein degradation and synthesis induced by different ligands of the estrogen receptor. We found that GREB1 is an HSP90 client which is regulated through degradation in response to ER modulation and identified off-target effects contributing to the pharmacological profile of raloxifene.

### Identification of Constitutive and Synthesis-Dependent HSP90 Clients

**Figure 5:**
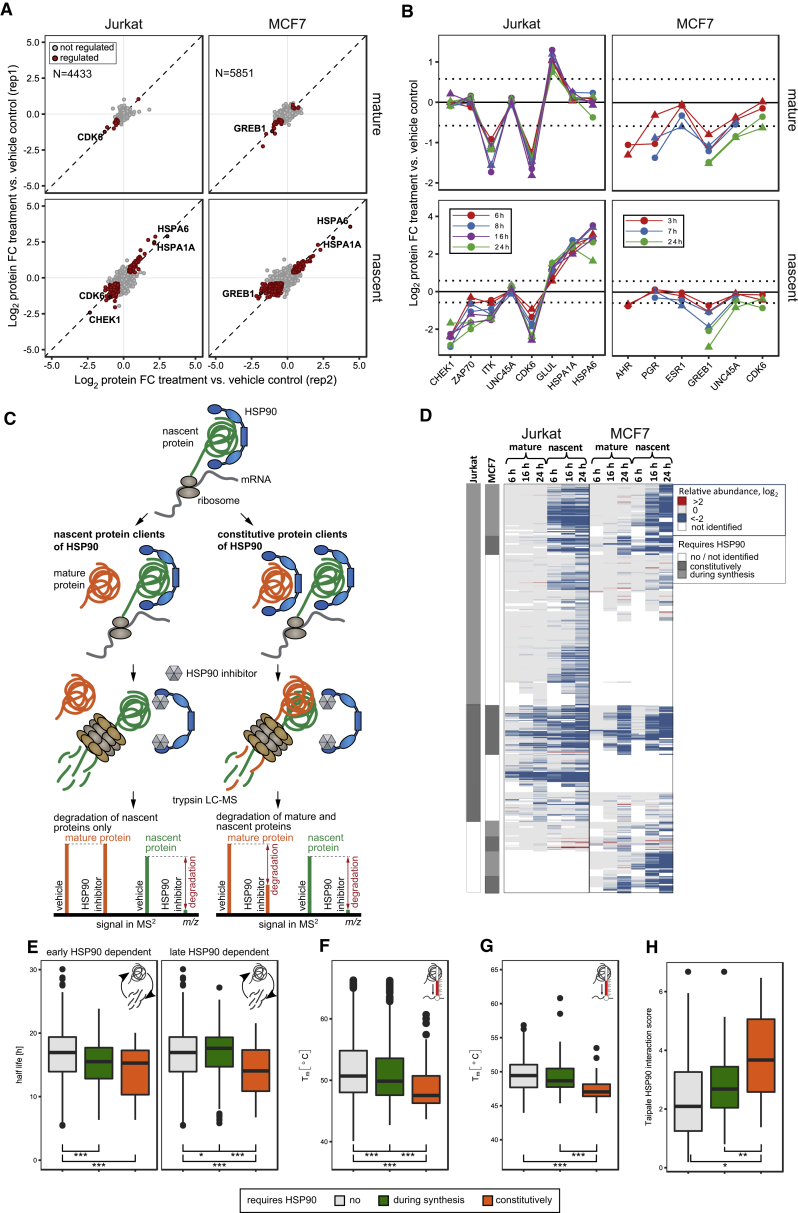
mPDP Identifies Proteins Constitutively Dependent on HSP90 Activity and Proteins Requiring HSP90 Activity Only during Synthesis (A) Scatterplots of protein fold changes (FC) for mature (upper panels) and nascent (lower panels) forms of proteins in Jurkat (left panels) and MCF-7 cells (right panels) treated with the HSP90 inhibitor 17-AAG for 6 and 7 hr, respectively, relative to vehicle-treated cells. Red closed circles indicate statistically significant regulation (p < 0.05), and the dashed diagonal is the equality line. N is the number of proteins robustly quantified in both replicates. (B) Line charts with markers of protein fold changes for in mature (upper panels) and nascent (lower panels) forms of selected proteins after treatment of Jurkat (left panels) and MCF-7 cells (right panels) with the HSP90 inhibitor 17-AAG relative to vehicle. Triangles and circles represent biological replicates 1 and 2, and colors indicate different treatment times. (C) Scheme for classification of HSP90-dependent proteins. (D) Heatmap representation of HSP90-dependent proteins identified using mPDP in combination with kinobeads enrichment. The color scheme indicates average fold changes of mature and nascent protein levels at different time points in Jurkat and MCF7 cells treated with 17-AAG relative to vehicle control from 2 biological replicates. Proteins classified as requiring HSP90 constitutively or during synthesis are indicated with the gray bar. Proteins not altered in abundance by 17-AAG treatment: gray; synthesis dependent: green, constitutively HSP90 dependent: orange. ^∗^p ≤ 0.05, ^∗∗^p ≤ 0.01, and ^∗∗∗^p ≤ 0.001 (E–H). Boxplots show protein half-lives of HSP90-dependent proteins in Jurkat cells. Early HSP90 dependent: 8-hr treatment with 17-AAG; late HSP90 dependent: 24 hr (E). Boxplots show protein thermal stabilities of non-kinase proteins in Jurkat cells grouped for their HSP90 dependence. Melting points (T_m_) were determined by TPP (F). Like in (F) but only for protein kinases (G). Boxplots show affinities of kinases for HSP90 as calculated by Taipale et al. (2012) and grouped by their HSP90 dependence (H). See also Tables S3 and S6 and Table S5.

**Figure S5:**
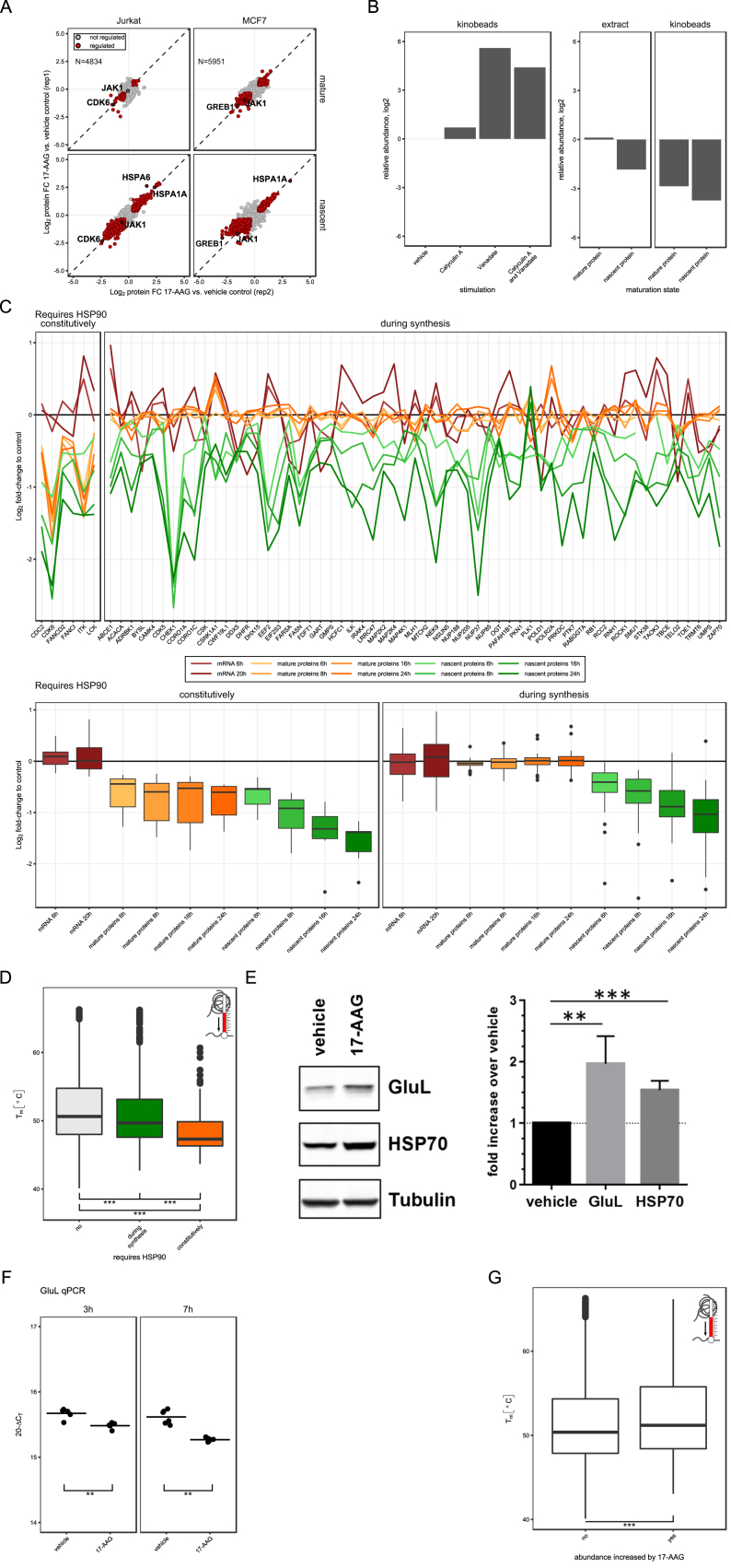
Validation of Effects Observed upon HSP90 Inhibition, Related to Figure 5 (A) Scatterplots showing protein fold changes (FC) observed in mature (upper panel) and nascent (lower panel) forms of proteins in Jurkat cells (left panel) and MCF-7 cells (right panel) treated for 24 h with 17-AAG relative to vehicle treated cells. Red closed circles indicate statistically significant regulation (p < 0.05) and the dashed diagonal indicates the equality line. N indicates the number of proteins robustly quantified in both replicates. (B) Protein fold change (FC) of ZAP70, following kinobeads affinity enrichment in cell extracts generated from Jurkat cells preincubated for 30 min with the phosphatase inhibitors calyculin A (0.05 μM) and orthovanadate (30 μM), individually or in combination (left panel) relative to vehicle treated cells. Protein fold change of mature and nascent ZAP70 after 24 h incubation with 17-AAG (central panel) and subsequent kinobeads affinity enrichment (right panel). (C) Comparison of relative mRNA levels as reported in Fierro-Monti et al. PloS ONE 2013 with the mPDP dataset of mature and nascent relative protein levels generated in Jurkat cells after treatment with 17-AAG at different time points for a subset of genes referred to in both datasets (upper panel). Boxplot representation showing log2 FC for mature, nascent protein and mRNAs (same subset displayed in the upper panel) for different 17-AAG treatments (lower panel). (D) Boxplots of proteins thermal stabilities in Jurkat cells (displayed as Tm in degrees Celsius) grouped according to HSP90 dependence, synthesis or constitutive (^∗^ p ≤ 0.05, ^∗∗^ p ≤ 0.01 and ^∗∗∗^ p ≤ 0.001). Melting points (T_m_) were determined by thermal proteome profiling. (E) Representative protein immunoblots of GLUL, HSP70 and Tubulin in Jurkat cells treated for 6 h with 10 μM 17-AAG. Bar chart: Quantification of band intensity from 3 independent experiments (mean with SD) displayed as fold increase normalized to control vehicle (^∗^ p ≤ 0.05, ^∗∗^ p ≤ 0.01 and ^∗∗∗^ p ≤ 0.001). (F) qPCR of GLUL mRNA levels by in Jurkat cells after treatment with 17-AAG (10 μM) for 3 and 7 hr. Data are displayed as 20-ΔCT (cycle times for GLUL amplification normalized to housekeeping gene RPL7) of two independent experiments performed in triplicate (^∗^ p ≤ 0.05, ^∗∗^ p ≤ 0.01 and ^∗∗∗^ p ≤ 0.001). (G) Boxplots of thermal stabilities (Tm) of proteins with significantly increased abundance by upon 17-AAG treatment in Jurkat cells (right) compared to proteins that do not change (left) at any measured time point (6-24 h). Only those proteins for which T_m_s could be determined are included (^∗∗∗^ p ≤ 0.001).

Next, we applied mPDP to stratify HSP90-dependent proteins based on whether they require HSP90 constitutively or only during synthesis. Jurkat and MCF-7 cells were treated with 10 μM 17-AAG, and samples were collected at different time points and processed as above (Figures 1, 5A, 5B, and S5A). Proteins with a significantly lower abundance in the mature protein pool in 17-AAG-treated cells compared to vehicle-treated cells were classified as constitutively requiring HSP90. In contrast, proteins that displayed a reduced abundance only in the nascent protein pool in 17-AAG-treated cells were classified as requiring HSP90 only during synthesis (Figure 5C). Since many known HSP90 clients are kinases (Sharma et al., 2012, Wu et al., 2012) additional kinobeads enrichment was performed to increase kinase coverage (Figure 5D). At early time points HSP90 inhibition caused depletion of mature and nascent CDK6 and tyrosine-protein kinases ZAP70, BLK, and ITK in Jurkat cells and the nuclear hormone receptors PGR, ESR1, and AHR in MCF-7 cells (Figure 5B), suggesting that these proteins constitutively require HSP90. This is in agreement with the known constitutive HSP90 dependence of these three nuclear hormone receptors, thus validating our approach (Echeverria and Picard, 2010). In contrast to kinobeads enriched ZAP70, analysis of the unfractionated proteome identified ZAP70 as HSP90 dependent only during synthesis (Figure S5B). Mimicking T cell receptor signaling in Jurkat cells by pervanadate treatment, the kinobeads enriched fraction of ZAP70 was greatly increased, suggesting preferential binding of the active kinase. Thus, active, but not inactive, ZAP70 is a constitutive HSP90 client.

For both cell lines, more than 1,000 proteins were downregulated in mature and nascent states across all time points, with 511 proteins being downregulated in both cell lines (Table S3). HSP90-dependent proteins with cell-type-specific expression included the tyrosine-protein kinase ITK in Jurkat cells, and GREB1 in MCF-7 cells (Wilhelm et al., 2014). Several proteins displayed differential HSP90 requirement in the two cell lines, e.g., CDK6 required HSP90 constitutively in Jurkat cells, but was not regulated significantly in MCF7 cells. Conversely, UNC45A a regulator of the progesterone receptor/hsp90 chaperoning pathway required HSP90 constitutively in MCF7 cells, but not in Jurkat cells (Figure 5B). For a large fraction of proteins only nascent forms were downregulated by 17-AAG suggesting that most HSP90 clients require HSP90 only during synthesis. We compared our list of HSP90-dependent proteins with a comprehensive dataset of proteins known to interact with HSP90, the Picard list (Echeverria and Picard, 2014) (Table S4). Among the ca. 300 Picard list proteins that were identified with each cell line there was a strong bias for constitutively HSP90-dependent proteins over synthesis-dependent proteins (Table S5). This is expected because of the transient interaction of HSP90 with synthesis-dependent clients. For example, the protein kinase CHEK1, a synthesis-dependent protein in our data, could not be co-purified with HSP90, but requires HSP90 for folding (Arlander et al., 2006, Hartson and Matts, 1994). A subset of the HSP90 synthesis-dependent proteins overlapped with a published transcriptomics dataset of 17-AAG treated Jurkat cells (Fierro-Monti et al., 2013) and mRNA levels of most of these proteins were not downregulated (Figure S5C), consistent with a posttranscriptional regulatory mechanism. Substantial overlap was observed between the Picard list (Table S4) and constitutively HSP90-dependent kinases (Jurkat, 64%; MCF7, 74%), in agreement with observations that kinase clients interact stronger with HSP90 than other clients (Taipale et al., 2012). For kinases requiring HSP90 only during synthesis the overlap was lower (Jurkat, 56%; MCF7, 43%).

In summary, mPDP identified many known HSP90 clients with a higher overlap being observed for proteins identified as constitutively dependent on the interaction with this chaperone.

Next, we mapped HSP90-dependent proteins to turnover data generated by dynamic SILAC in Jurkat cells and found that they have shorter half-lives than other proteins, with constitutively HSP90-dependent proteins having shorter half-lives than those that are synthesis-dependent (Figure 5E; Table S6). To probe if the constitutive HSP90 requirement and shorter half-lives indicate a less stable fold than other proteins, we mapped the mPDP data to proteome-wide thermal stability measurements (Savitski et al., 2014). We found that the thermal stability of HSP90-dependent proteins is lower than for other proteins, with constitutively HSP90 requiring proteins being less thermostable than those that are synthesis dependent. A comparable trend was observed when looking at protein kinases only (Figures 5F, 5G, and S5G; Table S6). Comparing our data to a study that determined binding affinities of HSP90 to kinases (Taipale et al., 2012), we found that constitutively HSP90 requiring kinases have higher binding affinities to HSP90 than synthesis clients (Figure 5H).

Treatment with 17-AAG also upregulated several nascent proteins, including the heat shock response proteins HSPA6 and HSPA1A (Figures 5A and 5B) and reduced degradation of glutamine synthetase (GLUL) (Figures 5B and S5E) consistent with previous reports (Arad et al., 1976) and unchanged mRNA levels (Figure S5F). Further, proteins upregulated upon HSP90 inhibition tend to have a more stable fold than other proteins (Figure S5G; Table S6).

### Immunomodulatory Mechanism of HSP90 Inhibition in T Cells

**Figure 6:**
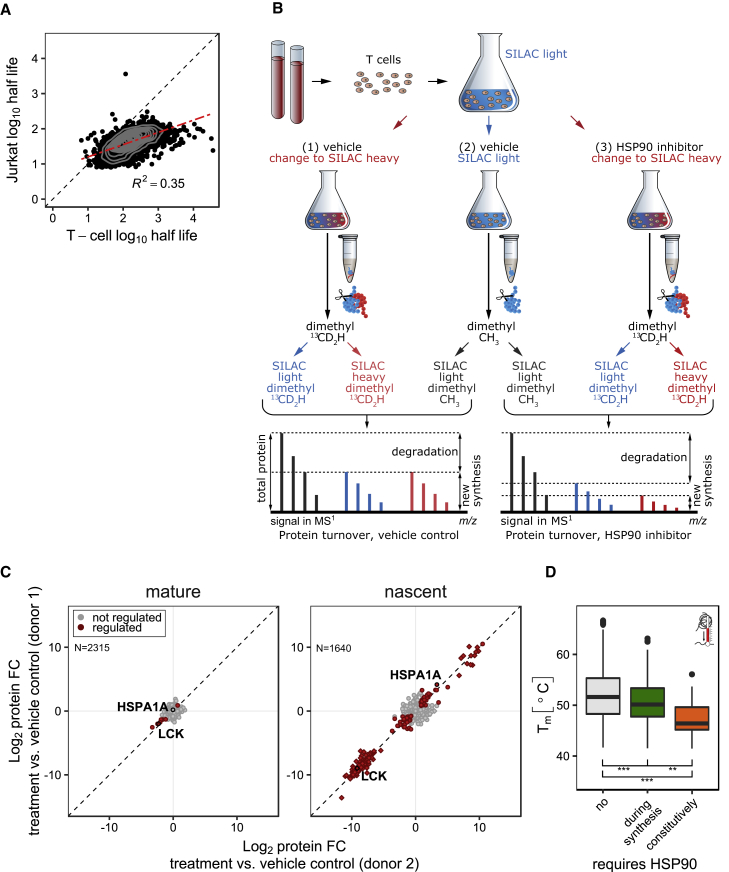
Profiling of HSP90-Dependent Proteins in T Cells (A) Correlation of protein half-lives in resting primary T cells and Jurkat cells after correction for cell division. R^2^ = 0.35. The dashed black line is the equality line and the linear fit (red) indicates a 2- to 3-fold higher protein turnover in Jurkat cells. (B) Schematic representation of the mPDP strategy for nondividing cells. (C) Scatterplots of fold changes for mature (left) and nascent (right) forms of proteins upon 24-hr 17-AAG treatment of primary T cells collected from two donors. Red color indicates statistically significant regulation (p < 0.05). Proteins marked as rectangles are quantified against background. The dashed diagonal is the equality line. (D) Boxplots show thermal stabilities of proteins in resting T cells that were either not HSP90 dependent (gray), synthesis dependent (green), or constitutively HSP90 dependent (orange). Melting points (T_m_) were determined by TPP in resting T cells of three donors. See also Tables S3 and S6.

**Figure S6:**
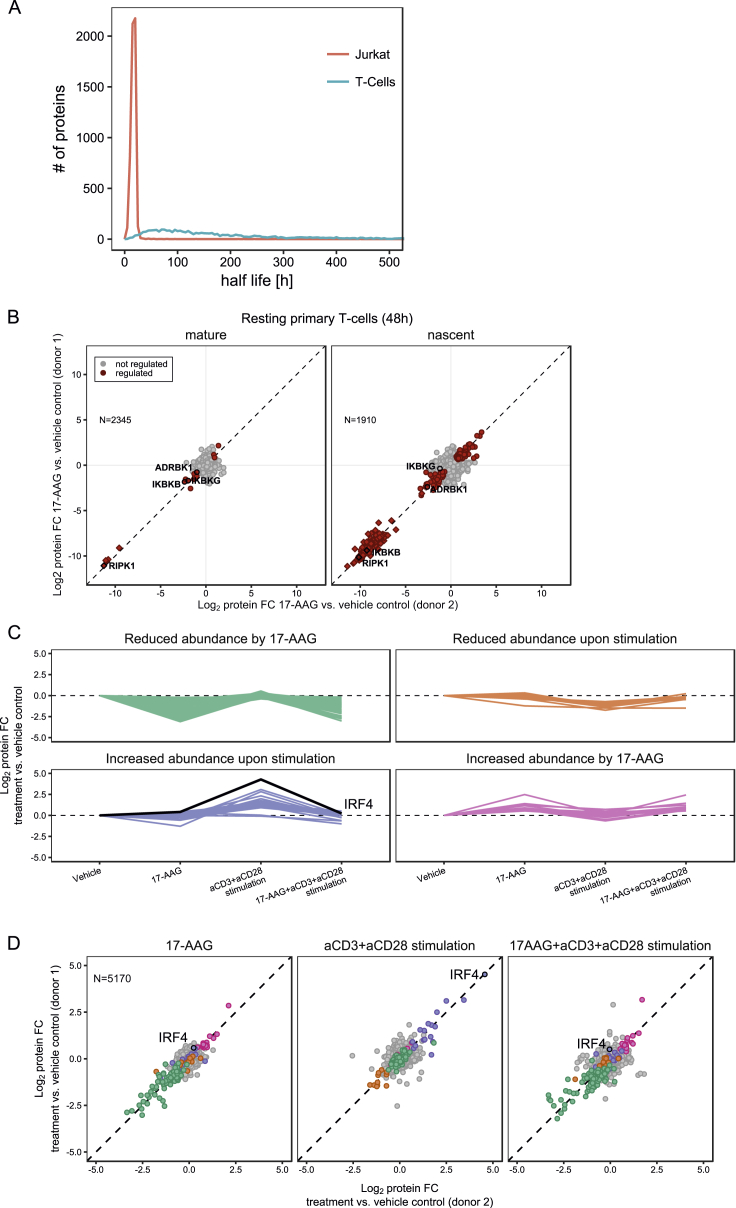
Effects of HSP90 Inhibition in Resting and Activated Primary T Cells, Related to Figure 6 (A) Display of protein half-lives (h) distribution in Jurkat and resting primary T cells, determined by dynamic SILAC labeling. Half-lives have not been corrected for the cell cycle time in Jurkat cells. (B) Scatterplots showing protein fold changes (FC) observed in mature (left panel) and nascent (right panel) forms of proteins in resting primary T cells treated for 48 h (lower panel) with 17-AAG relative to vehicle treated cells. Red closed circles indicate statistically significant regulation (p < 0.05), proteins in rectangle are quantified against background noise, and the dashed diagonal indicates the equality line. N indicates the number of proteins robustly quantified in both replicates. (C) K-means clustering of significantly regulated proteins from multiplexed expression proteomics analysis of T cells in response to 17-AAG treatment for 16 hr and subsequent TCR stimulation for 7 hr, averaged from two donors. Proteins significantly downregulated in response to 17-AAG are marked in green; upregulated proteins in magenta. Proteins significantly upregulated upon TCR stimulation are marked in blue, downregulated proteins are marked in orange. IRF4 is highlighted as indicator for TCR-mediated activation of T cells. (D) Scatterplots showing protein fold changes (FC) observed in multiplex expression proteomics analysis of T Cells in response to 17-AAG treatment for 16 hr and subsequent TCR stimulation for 7 hr, as described in (C). Proteins are colored according to the clusters from the K-means clustering in © and the dashed diagonal indicates the equality line. N indicates the number of proteins robustly quantified in both replicates.

Next, we investigated proteome homeostasis and HSP90-dependent proteins in resting, primary T cells isolated from healthy donors. In dynamic SILAC proteomics experiments, protein turnover was much lower in T cells than in Jurkat cells (Figure S6A), and, despite correction for the cell division rate, most proteins turned over 2–3 times faster in Jurkat cells (Figure 6A), suggesting a more stable proteome in primary T cells.

**Figure 7:**
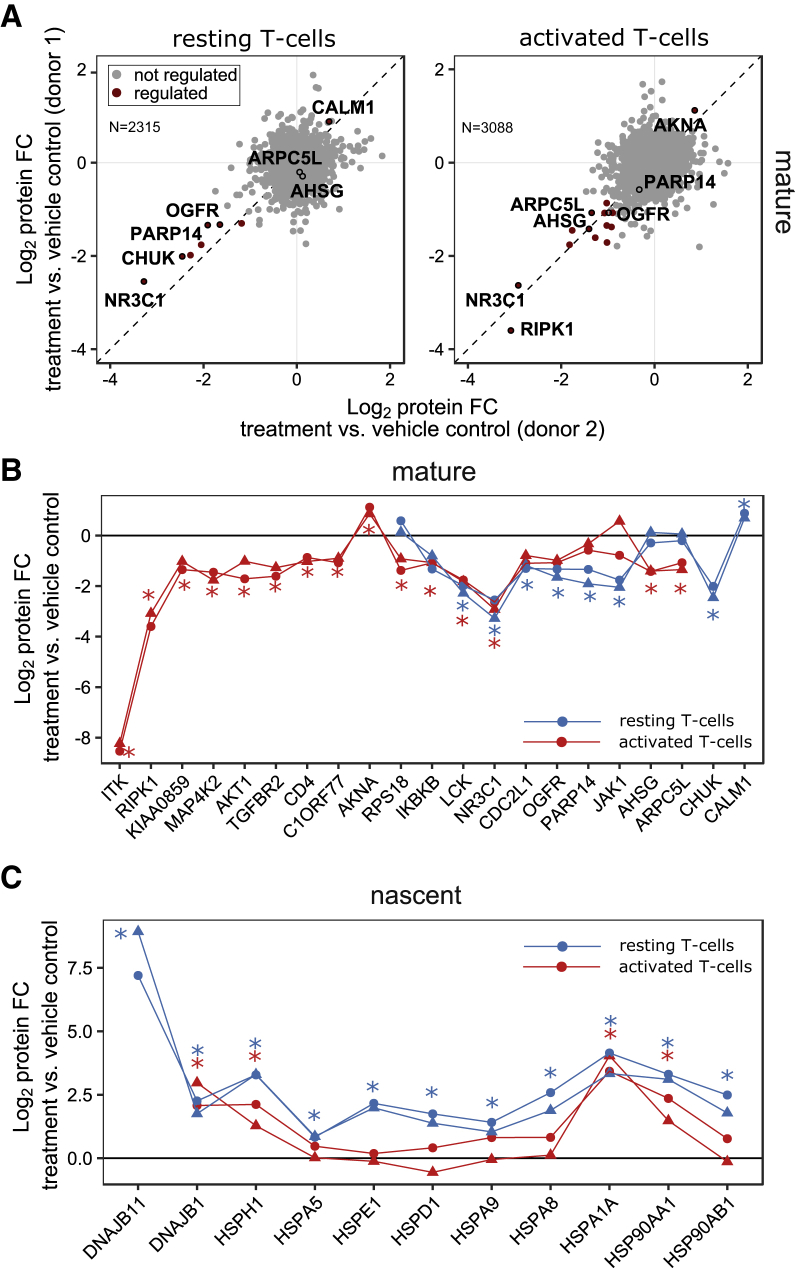
Comparison of HSP90-Dependent Proteins in Resting and Activated Primary T Cells (A) Scatterplots comparing global effects of HSP90 inhibition on mature proteins in resting (left) and anti-CD3/CD28 activated (right) primary T cells. Protein fold changes of 17-AAG versus vehicle treated samples are shown for two donors (full graph in Figure S7A). Significantly regulated proteins are marked in red (p < 0.05); the dashed line is the equality line. (B) Line chart with markers of fold changes for mature forms of selected proteins in resting (blue) and activated T cells (red) upon treatment with 17-AAG relative to vehicle control. (C) Line chart with markers of fold changes determined for nascent heat shock family members in resting (blue) and activated T cells (red) upon treatment with 17-AAG relative to vehicle control. Triangles and circles indicate the two donors. Significance is indicated by an asterisk (p < 0.05) (B and C). See also Table S3.

Since mPDP used for cell lines requires full incorporation of stable isotope-enriched amino acids and is not applicable to non-dividing primary T cells, we adapted the strategy and combined dynamic SILAC labeling with reductive dimethylation by stable isotope encoded formaldehyde (Boersema et al., 2009) (Figure 6B). T cells isolated from two donors were SILAC labeled in the presence of 10 μM 17-AAG or vehicle and analyzed by LC-MS/MS (Figure 6C). Consistent with Jurkat results, HSP90-dependent proteins (Table S3) had lower thermal stability in primary T cells with constitutively dependent proteins being less stable than synthesis-dependent proteins (Figure 6D; Table S6). Seven constitutively HSP90-dependent proteins were identified after 24 hr, including the known HSP90 interactors NR3C1, CHUK, JAK1, and LCK (Figures 7A and 7B; Tables S3 and S4) and ten more were identified after 48 hr including known, e.g., IKBKG and RIPK1 (Figure S6B) (Echeverria and Picard, 2014, Schoof et al., 2009, Yorgin et al., 2000) and previously undescribed clients such as PARP14 and the opioid growth factor receptor (OGFR). Calmodulin (CALM1), a known HSP90 interactor, was stabilized upon HSP90 inhibition (Figures 7A and 7B). In the nascent protein pool, we found 182 regulated proteins 24 hr after 17-AAG treatment (129 down, 53 up), of which 27 have been reported as HSP90 interactors (Tables S3 and S4).

We then investigated how the proteome dependency on HSP90 changed upon T cell activation. Proteomic analysis of stimulated cells showed strong upregulation of markers of T cell activation, such as IRF4 (Pernis, 2002), which was not observed in cells pre-treated with 17-AAG (Figures S6C and S6D). Thus, HSP90 inhibition completely ablated T cell receptor (TCR) signaling, consistent with degradation of key T cell signaling proteins, such as LCK.

**Figure S7:**
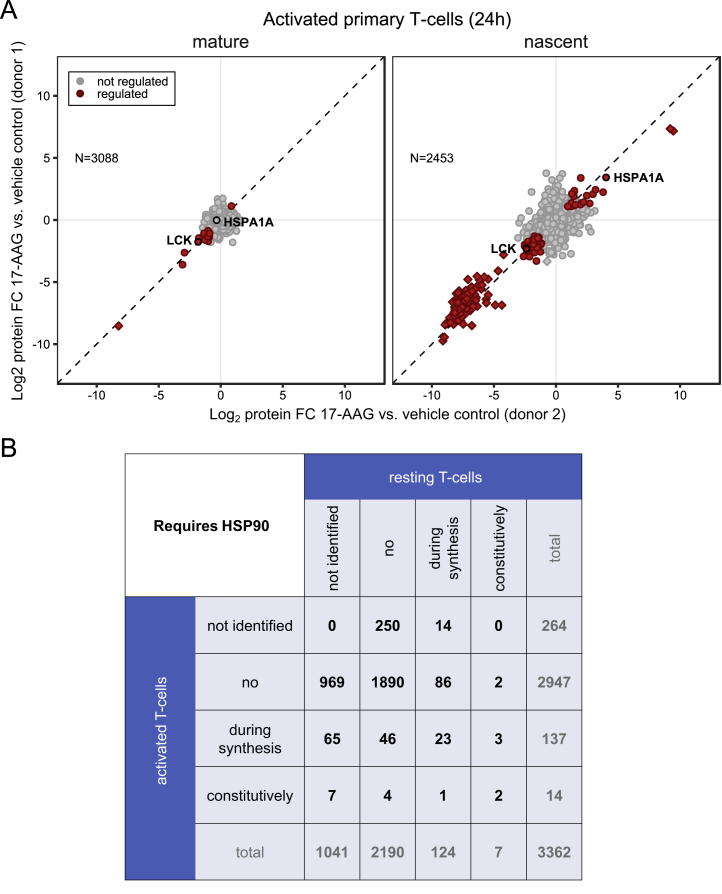
Comparison of HSP90 Inhibition Response in Resting and Activated T Cells, Related to Figure 7 (A) Scatterplots showing protein fold changes (FC) observed in mature (left panel) and nascent (right panel) forms of proteins in anti-CD3/CD28-activated primary T cells treated for 24 h with 17-AAG. Red closed circles indicate statistically significant regulation (p < 0.05), proteins in rectangle are quantified against background noise and the dashed diagonal indicates the equality line. N indicates the number of proteins robustly quantified in both replicates. (B) Comparison of proteome HSP90 dependence between resting and anti-CD3/CD28-activated primary T cells at 24 h. Proteins are grouped as follow: not identified, not regulated (no), dependent on HSP90 during synthesis, or constitutively. The number of proteins overlapping the different groups is indicated in a matrix display.

Next, T cells were first exposed to anti-CD3 and anti-CD28 for induction of the canonical TCR pathway for 14 hr followed by 17-AAG treatment for 24 hr and then processed with the mPDP workflow for non-dividing cells. In the nascent pool, we identified 164 regulated proteins (142 down and 22 up), of which 20 were previously described as HSP90 interactors (Echeverria and Picard, 2014) (Figure S7A; Tables S3 and S4) and differential induction of heat shock proteins in activated T cells versus resting T cells was observed (Figure 7C). A group of 14 constitutively HSP90-dependent proteins was discovered, of which eight proteins are on the Picard list (e.g., ITK, RIPK1, TGFBR2, and AKT1) and only NR3C1, IKBKB, and LCK were also detected as constitutively HSP90 dependent in resting T cells, at this time point (Figures 7A, 7B, and S6B). Levels of mature CD4 were also reduced upon 17-AAG treatment in agreement with previous reports indicating HSP90-dependent degradation (Bae et al., 2013). Among the five proteins with so far undescribed HSP90 dependence, AHSG, ARPC5L, and RPS18 were also identified but not regulated in resting T cells, indicating a conditional requirement for HSP90 for these proteins in the context of T cell activation (Figures 7A and 7B; Table S3). Further, AKNA, a nuclear repressor involved in immune responses (Siddiqa et al., 2001), was stabilized after 17-AAG treatment in activated T cells, but was not identified in resting T cells.

## Discussion

We have developed mPDP, a strategy that combines dynamic SILAC (Doherty et al., 2009, Schwanhäusser et al., 2011) with chemical labeling by tandem mass tags. Differentiating from previous work (Boisvert et al., 2012, Welle et al., 2016), mPDP analyzes multiple different treatment conditions in a single experiment, in full biological duplicates and thus enables identifying subtle effects on protein homeostasis. Relative quantification of proteins already synthesized when cells are perturbed and dynamic SILAC is started, allows for the sensitive identification of regulatory effects on protein degradation. In contrast, protein regulation only in the protein pool synthesized *de novo* after a cellular treatment is attributed to (post)transcriptional regulation.

First, we applied mPDP to study proteome effects of the bromodomain and extra-terminal domain (BET) family inhibitor JQ1 and a JQ1-PROTAC since mPDP enables distinguishing PROTAC-induced degradation of the BET proteins from BET-dependent transcriptional regulation. BET inhibitors, such as JQ1 and I-BET151, and JQ1-derived targeted degraders have been used widely as selective probes to investigate the roles of BRD2,3,4 and BRDT in transcriptional regulation and in disease mechanisms (Dawson et al., 2011, Filippakopoulos et al., 2010, Lu et al., 2015, Nicodeme et al., 2010, Winter et al., 2017). However, potential contributions of off-target activities to observed phenotypes must not be neglected as shown by the unexpected degradation of FYTTD1 by JQ1-VHL PROTAC. Previous studies indicated that the increased apoptosis upon treatment with JQ1 PROTAC compared to JQ1 inhibitor was caused by depletion of BET proteins in particular of BRD4 (Lu et al., 2015, Winter et al., 2017). Our data suggest that off-target binding of JQ1-based PROTACs to FYTTD1 and its subsequent degradation contributes to the observed complete arrest of protein synthesis. FYTTD1 is an adaptor of the TREX complex required for mRNA export from the nucleus to the cytoplasm (Hautbergue et al., 2009), and JQ1-VHL PROTAC, but not JQ1-Az, caused rapid degradation of this protein. Thermal proteome profiling revealed destabilization of FYTTD1 by JQ1 but not by the structurally different I-BET 151. Further, JQ1-VHL PROTAC, but not JQ1-Az or a PROTAC based on I-BET151, led to substantial RNA accumulation in the nucleus in THP-1 cells consistent with inactivation of the TREX complex. In addition, JQ1-VHL PROTAC led to depletion of RNA polymerase 2 with potential consequences on RNA synthesis. Pol2 inhibitors have been proposed as potential therapeutics for dormant leukemia cells (Pallis et al., 2013); it is tempting to speculate that the JQ1-VHL PROTAC described here might target these cells.

TPP revealed additional JQ1 off-targets including the sterol acylase SOAT1. Future studies using compounds with different off-target profiles will inform to what extent off-target inhibition of the atherogenic protein SOAT1 (Meiner et al., 1996) contributes to the anti-atherosclerotic efficacy of JQ1 (Brown et al., 2014). Interestingly, mPDP identified rapid degradation of the nuclear BET targets and FYTTD1 by JQ1-VHL PROTAC, but not of SOAT1, a transmembrane protein located in the ER. These findings underscore that degradation selectivity is defined not only by ligand affinities for the targets, but also by other factors, such as target localization.

In a second study, we investigated the effects of selective estrogen receptor modulators on protein homeostasis in MCF-7 cells and identified the estrogen responsive protein GREB1 as a constitutive HSP90 client whose degradation is indirectly induced upon ESR1 inhibition. mPDP further demonstrated different effects of the SERMs raloxifene and GW7604 on ESR1 stability consistent with a previous report (Wu et al., 2005) and identified differential effects on a subset of proteins indicating differences in the mechanisms of action of these two drugs, likely due to off-targets. For example, raloxifene treatment led to reduced degradation of the VPS34 inhibitor Rubicon, suggesting effects on autophagy consistent with the reported induction of cell death via an autophagy-dependent mechanism (Kim et al., 2015). Raloxifene also enhanced degradation of the transmembrane protein TMEM97, a regulator of cellular cholesterol homeostasis (Bartz et al., 2009), which suggests a mechanistic link to the hitherto unexplained effects of raloxifene on lipid and lipoprotein levels in postmenopausal women (Dayspring et al., 2006).

Third, we studied the HSP90-dependent proteome by mPDP. Previous studies investigating proteome-wide effects of HSP90 measured changes in protein abundances but did not discern between clients requiring permanent interaction with HSP90 and those only interacting with the chaperone during maturation (Fierro-Monti et al., 2013, Sharma et al., 2012, Wu et al., 2012). Our study distinguishes these HSP90 dependencies in a global analysis and we discovered that constitutively HSP90-dependent proteins have significantly lower half-lives and lower fold stabilities compared to those requiring HSP90 only during synthesis, extending previous findings that HSP90 client kinases tend to have a lower fold stability than non-clients (Taipale et al., 2012). Furthermore, we found that constitutively HSP90-dependent kinases bind to HSP90 with significantly higher affinities than kinases requiring HSP90 only during synthesis. Taken together, our study identified that constitutive HSP90 dependence of proteins is more widespread than previously appreciated and comprises proteins that are characterized by low-thermal stability and rapid turnover. Further, many proteins displayed a cell-line-dependent requirement for HSP90.

The adaptation of mPDP to primary cells allowed the proteomic analysis of mature and nascent HSP90 clients in resting and TCR-activated human T cells and identified several key TCR-signaling intermediates as constitutively HSP90 dependent. For calmodulin (CALM1), a primary cellular calcium sensor, 17-AAG treatment induced stabilization indicating a direct role of HSP90 for its proteasomal degradation in agreement with previous observations in cell-free systems (Whittier et al., 2004). We also found several constitutively HSP90-dependent proteins that have not been previously described as HSP90 clients including Fetuin-A (AHSG), a mineral carrier protein involved in the formation and clearance of calciprotein particles (CPPs). The rapid cellular Ca^2+^ influx resulting from TCR stimulation, might lead to the formation of CPPs which have been reported to contain HSP90 (Ho et al., 2012). We therefore speculate that the stability of Fetuin-A is regulated by HSP90 activity exclusively upon formation of the CPP complexes following TCR stimulation. Finally, the immunosuppressive nuclear factor AKNA (Ma et al., 2011) showed increased levels in 17-AAG treated, stimulated T cells, thus suggesting a role for HSP90 in targeting AKNA for degradation. These data open new perspectives for strategies aiming at the restoration of AKNA function in the context of inflammation and cancer (Moliterno and Resar, 2011).

In conclusion, we have established mPDP as a powerful means to elucidate the mechanism of action of bioactive molecules, thus complementing current proteome-wide target identification strategies. mPDP identifies targets of protein degraders and, more generally, enables the discovery of regulatory degradation mechanisms in biological systems.

## STAR★Methods

### Key Resources Table

**Table:** 

REAGENT or RESOURCE	SOURCE	IDENTIFIER
**Antibodies**		

anti-CD3	Abcam	Cat# ab8090
anti-CD28	BD Biosciences	Cat# 555725
GREB1	Abcam	Cat# ab72999
GLUL	BD Biosciences	Cat# 610517
HSP70	Cell Signaling	Cat# 4872
α-tubulin	Sigma	Cat# T6793
IRDye800 (anti-mouse)	Li-Cor	Cat# 925-32210
IRDye680 (anti-rabbit)	Li-Cor	Cat# 925-68071

**Biological Samples**		

Human buffy coats (consented donors, IRB approved)	DRK-Blutspendedienst Mannheim	N/A

**Chemicals, Peptides, and Recombinant Proteins**		

17-AAG	LC LABS	Cat# A-6880
I-BET151	GSK compound collection (Dawson et.al. 2011)	CAS: 1300031-49-5
estradiol	Sigma-Aldrich	Cat# E8875; E2758
GW7604	GSK compound collection	CAS: 195611-82-6
raloxifene	GSK compound collection	CAS: 84449-90-1
JQ1	Carbosynth	Cat# FC43273
calyculin A	Apollo Scientific	Cat# BIC1019
sodium orthovanadate	Sigma-Aldrich	Cat# S6508
JQ1-VHL PROTAC	This publication	N/A
JQ1-Az	This publication	N/A
VHL alkyne	This publication	N/A
IBET-151-PROTAC	This publication	N/A
TMT10plex	ThermoFisherScientific	Cat# 90406
Basal RPMI medium	GIBCO	Cat# A24942-01
Dialysed FBS	GIBCO	Cat# 26400-044
Glucose 2g/L	GIBCO	Cat# A24940-01
L-Glutamine (200 mM)	GIBCO	Cat# 24030-032
L-Arginine light	Sigma	Cat# A8094-25G
L-Lysine light	Sigma	Cat# L9037-25G
L-Arginine heavy (SILAC)	Thermo	Cat# 88434
L-Lysine heavy (SILAC)	Sigma	Cat# 608041-1G
Cy3-Oligo(dT)50	Sigma	N/A
Wheat Germ Agglutinin (WGA) Alexa Fluor 647 Conjugate	Life Technologies	Cat# W32466
Hoechst 33258	Sigma	Cat# H3569
Recombinant FYTTD1	Abcam	Cat# ab164533
Protein G beads	Sigma	Cat# P7700
benzonase	Sigma	Cat# E1014
Igepal CA-630	Sigma	Cat# I8896

**Critical Commercial Assays**		

T cells negative selection kit	StemCell Technologies	Cat# 19051
CellTiter-Glo	Promega	Cat# G9242
RNAquous extraction kit	Life Technologies	Cat# Q10212
Maxima First Strand cDNA kit	Thermo Fisher	Cat# K1641
Qubit kit	Life Technologies	Cat# Q10212
TaqMan master mix	Thermo Fisher	Cat# 4369016

**Deposited Data**		

Supplemental datasets for individual Figures	Mendeley Data	https://doi.org/10.17632/8pzhg2tdyb.1
Multiplexed proteome dynamics profiling of JQ1-PROTAC	PRIDE	PXD008637
Thermal proteome profiling of JQ1	PRIDE	PXD008638
2 Dimensional Thermal proteome profiling of JQ1	PRIDE	PXD008626
Multiplexed proteome dynamics profiling of Estradiol and SERMs	PRIDE	PXD008636
Expression proteomics combination treatment of Estradiol and SERMs	PRIDE	PXD008628
Multiplexed proteome dynamics profiling of HSP90 inhibitor 17-AAG	PRIDE	PXD008633
Immunoaffinity enrichment of GREB1 to co-enrich HSP90	PRIDE	PXD008631
Thermal proteome profiling of Jurkat cells	PRIDE	PXD008639
Kinobeads affinity enrichment from lysate derived from stimulated cells	PRIDE	PXD008632
Jurkat protein half live	PRIDE	PXD008629
T cell protein half live	PRIDE	PXD008630
Multiplexed proteome dynamics profiling of HSP90 inhibitor 17-AAG in T cells	PRIDE	PXD008635
Multiplexed proteome dynamics profiling of HSP90 inhibitor 17-AAG in anti-CD3 and anti-CD28 stimulated T cells	PRIDE	PXD008634
Expression profiling of HSP90 inhibitor 17-AAG in anti-CD3 and anti-CD28 stimulated T cells	PRIDE	PXD008627

**Experimental Models: Cell Lines**		

THP-1	ATCC	TIB-202
MCF-7	ATCC	HTB-22
Jurkat	ATCC	TIB-152

**Oligonucleotides**		

Amplicon context sequence for GLUL (FAM)	Thermo Fisher	Cat# 4331182
ACCCGGACCCTGGACA GTGAGCCCAAGTGT GTGGAAGAGT		Assay ID# Hs00365928
Amplicon context sequence for RPL7 (VIC)	Thermo Fisher	Cat# 4331182
AGAACCCAAATTGGC GTTTGTCATCAGAATCAGAGGTA		Assay ID# Hs02596927

**Software and Algorithms**		

ImageJ 1.45 s	NIH	https://imagej.nih.gov/ij/download.html
CellProfiler 2.1.1	Broad Institute	http://cellprofiler.org
Mascot 2.4	Matrix Science, Boston, MA	N/A
isobarQuant	Franken et al., 2015	https://github.com/protcode/isob/archive/1.1.0.zip
R 3.4.1	R Core Team	https://www.R-project.org
TPP library	Franken et al., 2015	http://bioconductor.org/biocLite.R; library(“TPP”)
Spotfire 7.0.1.24	Tibco	https://spotfire.tibco.com
Graphpad Prism 7	GraphPad Software	https://www.graphpad.com

### Contact for Reagent and Resource Sharing

Further information and requests for resources and reagents should be directed to and will be fulfilled by the Lead Contact, Marcus Bantscheff (marcus.x.bantscheff@gsk.com).

### Experimental Model and Subject Details

#### Cell culture

##### Jurkat cells

Jurkat cells (ATCC TIB-152, male) were cultured in RPMI-based SILAC-L and SILAC-H medium in parallel for 14 days, and incorporation of heavy isotopes was verified by mass spectrometry. For multiplexed proteome dynamics profiling, cells were re-suspended in opposite SILAC label medium, incubated in presence of 17-AAG (10 μM) or vehicle (DMSO) for the indicated time periods at 37°C, 5% CO_2_, washed, pelleted, and snap-frozen in liquid N_2_. No impact on cell viability was observed (ATP-CellTiterGlo, Promega, data not shown).

In activation experiments, Jurkat cells were exposed to calyculin A (50 nM, Apollo Scientific) and sodium orthovanadate (30 μM) for 30 min and then harvested as described above.

##### THP-1 cells

THP-1 cell cultures (ATCC TIB-202, male) were established in normal growth medium (RPMI1640 + 10% FBS) or RPMI-based SILAC-L and SILAC-H medium. Cells were seeded in opposite SILAC label medium or normal growth medium and incubated in presence of compounds (1 μM and 10 μM JQ1-VHL PROTAC, 10 μM JQ1-Az, and 10 μM VHL alkyne) or vehicle (DMSO) for the indicated time points (6 or 24 h) at 37°C, 5% CO_2_. For harvesting, the cells were washed in PBS, pelleted, and snap-frozen in liquid N_2_.

This cell line has been authenticated using the Promega GenePrint10 kit to generate a STR Profile for comparison to the expected profile reported by ATCC. The STR Profile generated matches exactly to the expected ATCC STR Profile.

##### MCF-7 cells

MCF-7 cells (ATCC HTB-22, female) were cultured in RPMI-based SILAC-L and SILAC-H medium supplemented with non-essential amino acids (NEAA, 1x, Thermo Fisher) and sodium pyruvate (1 mM, Thermo Fisher) but without phenol red in parallel for 14 days, and incorporation of heavy isotopes was verified by mass spectrometry. For proteomic analysis, near-confluent (∼80%) cells were pulse labeled with ‘opposite’ SILAC label medium for 30 mins until compounds (10 μM 17-AAG, 0.4 μM GW7604, 0.4 μM raloxifene) and 0.4 μM estradiol were added. Cells were incubated for the indicated time points (3, 7 or 24 h) at 37°C, 5% CO_2_ without impact on cell viability (ATP, data not shown). In combination treatments, estradiol (0.1 μM) was added 15 min after selective estrogen receptor modulator (SERM) compound treatment (0.4 μM GW7604, 0.4 μM raloxifene) with a total incubation time of 24 h. For harvesting, the cells were scraped into cold PBS, washed, and snap-frozen in liquid N_2_.

This cell line has been authenticated using the Promega Cell ID System to generate STR Profiles for comparison to the expected profile reported by ATCC. The STR Profile generated matches exactly to the expected ATCC STR Profile.

##### T cells

Ethics statement: The use of buffy coat-derived peripheral blood mononuclear cells (PBMC) was reviewed and approved by the Ethical Committee of the Landesärztekammer Baden-Württemberg, Germany (#2007-036-f), based on the principles in the Declaration of Helsinki. Venous blood was collected from consented healthy adult volunteers. Pseudonymized buffy coats were obtained from the German Red Cross, Mannheim, and gender/sex, age were not disclosed.

Primary human CD3+ T cells were isolated by negative selection (StemCell Technologies) from PBMCs derived from buffy coats and accustomed to SILAC light medium (4 h, 37°C, 5% CO_2_). For proteome dynamic analysis of T cell activation (7.5 × 10^6^ cells/data point), cells were given into washed T-flasks pre-coated with anti-CD3 (Abcam, 1.5 μg/mL), supplemented with soluble anti-CD28 (3 μg/mL, BD Biosciences), and cultured for 14 h. Then, cells were pulse-labeled by transfer into SILAC heavy medium, re-stimulated with anti-CD3/anti-CD28, cultured for 1.5 h (lag time), and exposed to 17-AAG (10 μM) or vehicle (DMSO) for 24 h. Resting T cells were incubated similarly (for 24 h or 48 h) but without exposure to anti-CD3/anti-CD28, and the lag time between pulse labeling and 17-AAG/vehicle treatment was 4 h. For expression proteomic analysis of activated T cells (5 × 10^6^ cells/data point), freshly purified cells were adapted to SILAC light medium for 4 h, then treated with 17-AAG or vehicle for 16 h and transferred into washed T-flasks pre-coated with anti-CD3 and supplemented with anti-CD28 as described above. Cells were further incubated for 7 h, washed, pelleted, and snap-frozen in liquid N_2_. The viability of resting and anti-CD3/anti-CD28-activated T cells treated with 17-AAG or vehicle was not significantly altered after up to 48 h of culture. The ATP levels of vehicle- anti-CD3/anti-CD28-treated cells were significantly increased compared to resting cells, indicating successful T cell activation (data not shown). Experiments were performed on two individual donors and no statistical assessment of the sample size was done.

### Method Details

#### Reagents

Compounds JQ1, I-BET151, estradiol, 17-AAG, GW7604 and raloxifene were either available from the GSK compound collection or sourced commercially with purity of 98% or higher. Reagents and media were purchased from Sigma (St. Louis, MO) unless otherwise noted. SILAC “light” (L) medium for Jurkat and primary human T cells was made of basal SILAC RPMI medium supplemented with 10% dialysed fetal bovine serum (FBS), glucose (2 g/L), L-glutamine (2 mM), phenol red (all Thermo Fisher) as well as L-Arginine and L-Lysine (100 mg/L, each, Thermo Fisher). The corresponding SILAC “heavy” (H) medium contained stable isotope-labeled ^13^C_6_^15^N_2_-L-Lysine and ^13^C_6_^15^N_4_-L-Arginine (Thermo Fisher), both at 100 mg/L, instead of the “light” amino acids.

##### Chemical synthesis

All commercial solvents, reagents and building blocks are of reagent grade and were used as received without further purification unless otherwise specified. For separation of dichloromethane and aqueous layers during sample workup Radleys Phase Separation 6 mL Columns (RR99821) were used. Flash column chromatography was performed using a Biotage Isolera four purification system using Biotage flash silica cartridges. Preparative mass directed high performance liquid chromatography (preparative HPLC) was done on a XBridge BEH C18 OBD 5μm Prep Column (19 × 150 mm) at a flow rate of 30 mL/min, eluting with acetonitrile in water (0.2% formic acid as modifier). Purifications were conducted on a Waters autopurification system [detectors: 3100 Mass Detector and a 2996 Photodiode Array Detector]. Analytical UPLC (LCMS Method A) was done on a Waters Acquity BEH C18 1.7 μm Column (2.1 × 50 mm) at 40°C and a flow rate of 0.5 mL/min with a linear gradient over 4 min (5 to 100% acetonitrile in water, 0.2% formic acid as modifier). The instrument used for analysis was a Waters Acquity system [detectors: Acquity SQD and Acquity PDA]. Analytical UPLC (LCMS Method B) was done on a Waters Acquity BEH C18 1.7 μm Column (2.1 × 50 mm) at 40°C and a flow rate of 1 mL/min with a linear gradient over 1.5 min (3 to 100% acetonitrile in water, 0.1% formic acid as modifier). The instrument used for analysis was a Waters Acquity system [detectors: Acquity ZQ, Acquity ELSD, Acquity PDA]. The purity of all final compounds was 95% or higher as determined by LCMS (UV_254_). Proton (^1^H) and carbon (^13^C) NMR spectra were recorded on a Bruker Avance spectrometer (400 MHz) using the indicated deuterated solvent. Chemical shifts are given in parts per million (ppm) (δ relative to residual solvent peak for ^1^H and the central peak of the carbon multiplet of the deuterated solvent).

##### N-(2-(2-azidoethoxy)ethyl)-2-((6S)-4-(4-chlorophenyl)-2,3,9-trimethyl-6H-thieno[3,2-f][1,2,4]triazolo[4,3-a][1,4]diazepin-6-yl)acetamide (JQ1-Az):

**Figure:**
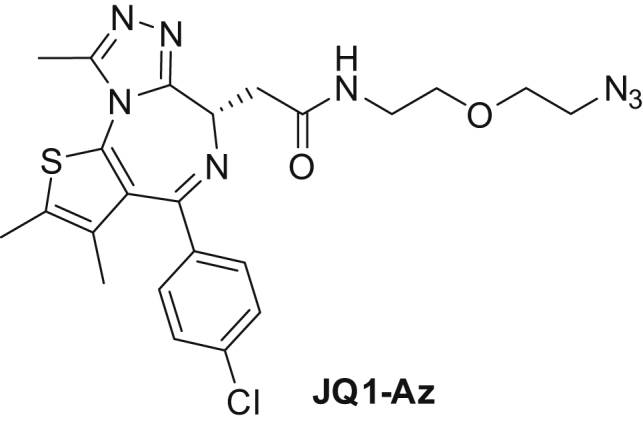

2-((6*S*)-4-(4-Chlorophenyl)-2,3,9-trimethyl-6*H*-thieno[3,2-*f*][1,2,4]triazolo[4,3-*a*][1,4]diazepin-6-yl)acetic acid (100 mg, 0.249 mmol) and 2-(2-azidoethoxy)ethanamine (35.7 mg, 0.274 mmol) were dissolved in 2 mL DMF at room temperature. Then DIPEA (0.131 mL, 0.748 mmol) was added, followed by HATU (142 mg, 0.374 mmol). The reaction mixture was stirred for 16 hr and then diluted with ethyl acetate (25 mL). This was then washed with 25 mL each of water (twice) and Brine. The organic layer was dried over sodium sulfate, filtered and evaporated. Flash chromatography (cyclohexane to ethyl acetate to 20% methanol in ethyl acetate) gave JQ1-Az as an off-white solid (92.6 mg, 72% yield). ^1^H NMR (400 MHz, DMSO-*d*_6_) δ 8.29 (t, *J* = 5.7 Hz, 1H), 7.49 (d, *J* = 8.4 Hz, 2H), 7.42 (d, *J* = 8.4 Hz, 2H), 4.51 (dd, *J* = 7.9, 6.1 Hz, 1H), 3.62 (t, *J* = 4.9 Hz, 2H), 3.50 (t, *J* = 5.9 Hz, 2H), 3.41 (t, *J* = 4.9 Hz, 2H), 3.35–3.16 (m, 4H), 2.59 (s, 3H), 2.41 (s, 3H), 1.62 (s, 3H). ^13^C NMR (101 MHz, DMSO-*d*_6_) δ 169.69, 162.98, 155.09, 149.79, 136.76, 135.20, 132.26, 130.68, 130.13, 129.81, 129.55, 128.44, 69.00, 68.97, 53.82, 49.95, 38.53, 37.52, 14.03, 12.67, 11.28. LCMS Method A (Rt = 2.43 min) *m/z* 513 [M+1]^+^.

##### (2S,4R)-1-((S)-3,3-dimethyl-2-(3-(prop-2-yn-1-yloxy)propanamido)butanoyl)-4-hydroxy-N-(4-(4-methylthiazol-5-yl)benzyl)pyrrolidine-2-carboxamide (VHL-alkyne):

**Figure:**
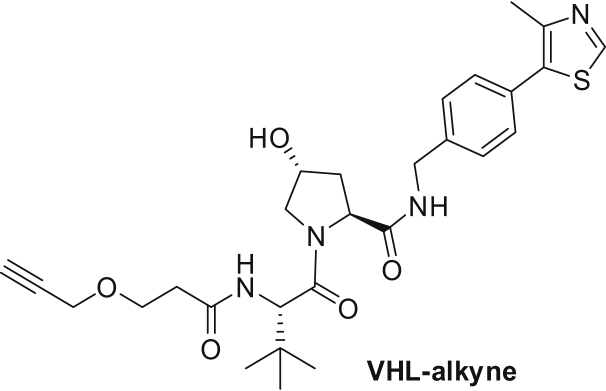

(2*S*,4*R*)-1-((*S*)-2-amino-3,3-dimethylbutanoyl)-4-hydroxy-*N*-(4-(4-methylthiazol-5-yl)benzyl)pyrrolidine-2-carboxamide hydrochloride (100 mg, 0.214 mmol) and 2,5-dioxopyrrolidin-1-yl 3-(prop-2-yn-1-yloxy)propanoate (48.2 mg, 0.214 mmol) were dissolved in 1 mL DMF at room temperature. Then triethylamine (0.030 mL, 0.214 mmol) was added. The reaction mixture was stirred for 3 hr and then directly purified by mass directed preparative HPLC. This gave VHL-alkyne as a white solid (79.7 mg, 69% yield). ^1^H NMR (400 MHz, Methanol-*d*_4_) δ 8.88 (s, 1H), 7.51–7.38 (m, 4H), 4.65 (s, 1H), 4.60–4.48 (m, 4H), 4.36 (d, *J* = 15.5 Hz, 1H), 4.16 (dd, *J* = 2.4, 0.8 Hz, 2H), 3.90 (dt, *J* = 11.1, 1.7 Hz, 1H), 3.84–3.72 (m, 3H), 2.85 (t, *J* = 2.4 Hz, 1H), 2.60 (ddd, *J* = 15.1, 7.5, 5.4 Hz, 1H), 2.53–2.44 (m, 4H), 2.22 (ddt, *J* = 13.1, 7.7, 2.0 Hz, 1H), 2.08 (ddd, *J* = 13.3, 9.1, 4.6 Hz, 1H), 1.04 (s, 9H). ^13^C NMR (101 MHz, Methanol-*d*_4_) δ 174.46, 173.49, 172.20, 152.84, 149.05, 140.28, 133.35, 131.53, 130.38, 128.98, 80.42, 76.05, 71.09, 66.92, 60.82, 59.03, 58.91, 57.99, 43.71, 38.91, 37.12, 36.72, 27.02, 15.79. LCMS Method A (Rt = 1.84 min) *m/z* 541 [M+1]^+^.

##### (2S,4R)-1-((2S)-2-(3-((1-(2-(2-(2-((6S)-4-(4-chlorophenyl)-2,3,9-trimethyl-6H-thieno[3,2-f][1,2,4]triazolo[4,3-a][1,4]diazepin-6-yl)acetamido)ethoxy)ethyl)-1H-1,2,3-triazol-4-yl)methoxy)propanamido)-3,3-dimethylbutanoyl)-4-hydroxy-N-(4-(4-methylthiazol-5 -yl)benzyl)pyrrolidine-2-carboxamide (**JQ1-VHL-PROTAC**):

**Figure:**
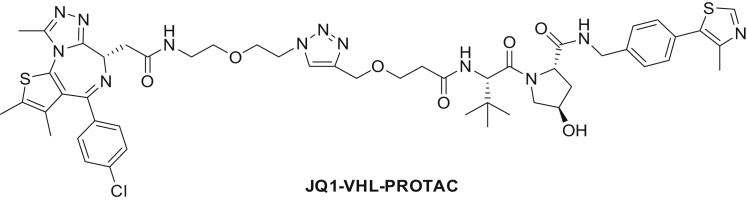

JQ1-Az (50 mM in DMSO, 234 μL, 0.012 mmol) and VHL-alkyne (50 mM in DMSO, 234 μL, 0.012 mmol) were mixed, dilute with 409 μL *tert*-butanol and then a premixed solution of tris(3-hydroxypropyltriazolylmethyl)amine (THPTA, 50 mM in water, 468 μL, 0.023 mmol) and copper (II) sulfate (100 mM in water, 117 μL, 0.012 mmol) was added. At last, a freshly prepared solution of sodium L-ascorbate (200 mM in water, 293 μL, 0.059 mmol) was added and the reaction mixture was stirred at room temperature overnight. The solution was diluted with water (5 mL) and extracted with 5 mL each of dichlormethane (twice) and ethyl acetate (twice). The organic layers were dried using a hydrophobic frit and sodium sulfate, combined and evaporated. Flash chromatography (0%–20% MeOH in dichloromethane) gave JQ1-VHL-PROTAC as a white solid (10.0 mg, 81% yield). ^1^H NMR (400 MHz, Chloroform-*d*) δ 8.71 (s, 1H), 7.98 (s, 1H), 7.63–7.55 (m, 2H), 7.46–7.29 (m, 9H), 4.76 (t, *J* = 8.2 Hz, 1H), 4.70–4.47 (m, 7H), 4.46–4.41 (m, 1H), 4.35 (dd, *J* = 15.1, 5.4 Hz, 1H), 4.15 (d, *J* = 11.4 Hz, 1H), 3.89–3.71 (m, 3H), 3.70–3.63 (m, 1H), 3.59 (dd, *J* = 11.3, 3.3 Hz, 1H), 3.54–3.22 (m, 6H), 2.65 (s, 3H), 2.59–2.47 (m, 4H), 2.46–2.32 (m, 4H), 2.19 (dd, *J* = 13.4, 7.9 Hz, 1H), 1.65 (s, 3H), 0.96 (s, 9H). LCMS Method A (Rt = 2.32 min) *m/z* 1053.5 [M+1]^+^.

##### 3-(2-azidoethoxy)-N-(2-(7-(3,5-dimethylisoxazol-4-yl)-8-methoxy-2-oxo-1-((R)-1-(pyridin-2-yl)ethyl)-1H-imidazo[4,5-c]quinolin-3(2H)-yl)ethyl)propanamide (IBET151-Az):

**Figure:**
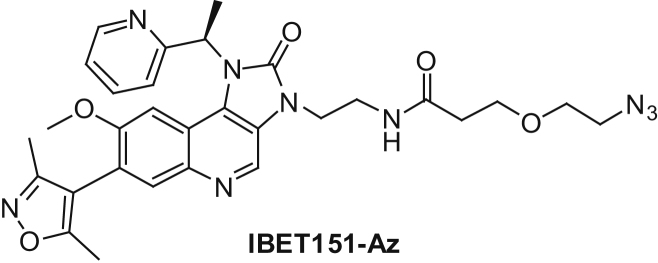

7-(3,5-dimethylisoxazol-4-yl)-8-methoxy-1-((*R*)-1-(pyridin-2-yl)ethyl)-1*H*-imidazo[4,5-*c*]quinolin-2(3*H*)-one (IBET151, 240 mg, 0.578 mmol) was dissolved in DMF (10 mL) and 2-*tert*-butylimino-2-diethylamino-1,3-dimethylperhydro-1,3,2-diazaphosphorine (polymer-bound, 1.00 g, ∼2.25 mmol) was added, then the mixture was stirred for 30 min before addition of 2-(2-bromoethyl)isoindoline-1,3-dione (147 mg, 0.578 mmol). The suspension was stirred for 6 hr and then poured onto a strong cation exchange cartridge (20 g, SCX-2). This was then washed with methanol (50 mL) and eluted with 2 M methanolic ammonia (50 mL). The eluant was evaporated. Flash chromatography (0%–10% 2 M methanolic ammonia in dichloromethane) gave 2-(2-(7-(3,5-dimethylisoxazol-4-yl)-8-methoxy-2-oxo-1-((*R*)-1-(pyridin-2-yl)ethyl)-1*H*-imidazo[4,5-*c*]quinolin-3(2*H*)-yl)ethyl)isoindoline-1,3-dione (260 mg, 76% yield) as pale yellow gum. This was used in the next step without any further purification. LCMS Method B (Rt = 0.92 min) *m/z* 589.4 [M+1]^+^.

2-(2-(7-(3,5-dimethylisoxazol-4-yl)-8-methoxy-2-oxo-1-((*R*)-1-(pyridin-2-yl)ethyl)-1*H*-imidazo[4,5-*c*]quinolin-3(2*H*)-yl)ethyl)isoindoline-1,3-dione (200 mg, 0.340 mmol) was dissolved in ethanol (10 mL) and hydrazine monohydrate (0.098 mL, 3.12 mmol) was added, then the mixture was heated at reflux under nitrogen for 2 hr. The mixture was cooled, diluted with water (20 mL) and evaporated to half volume. Then it was extracted with ethyl acetate (2 × 20 mL) and the combined organic layer was washed with brine, dried over sodium sulfate and evaporated. This gave 3-(2-aminoethyl)-7-(3,5-dimethylisoxazol-4-yl)-8-methoxy-1-((R)-1-(pyridin-2-yl)ethyl)-1H-imidazo[4,5-c]quinolin-2(3H)-one (122 mg, 78% yield) as colorless glass. ^1^H NMR (400 MHz, Chloroform-*d*) δ 8.74 (s, 1H), 8.66 (d, *J* = 4.7 Hz, 1H), 7.82 (s, 1H), 7.63 (dd, *J* = 8.4, 6.7 Hz, 1H), 7.32–7.21 (m, 2H), 6.85 (s, 1H), 6.47 (d, *J* = 7.4 Hz, 1H), 4.22 (t, *J* = 6.3 Hz, 2H), 3.54 (s, 3H), 3.25 (t, *J* = 6.3 Hz, 2H), 2.30 (s, 3H), 2.19–2.12 (m, 6H). LCMS Method B (Rt = 0.62 min) *m/z* 459.3 [M+1]^+^.

3-(2-aminoethyl)-7-(3,5-dimethylisoxazol-4-yl)-8-methoxy-1-((R)-1-(pyridin-2-yl)ethyl)-1H-imidazo[4,5-c]quinolin-2(3H)-one (30.0 mg, 0.065 mmol) and 2,5-dioxopyrrolidin-1-yl 3-(2-azidoethoxy)propanoate (16.8 mg, 0.065 mmol) were dissolved in DMF (1 mL) and then triethylamine (0.027 mL, 0.196 mmol) was added. The reaction mixture was stirred at room temperature for 3 hr. It was then directly purified by mass directed preparative HPLC. This gave IBET151-Az (27.9 mg, 0.046 mmol, 71% yield) as a white solid. ^1^H NMR (400 MHz, Methanol-*d*_4_) δ 8.82 (s, 1H), 8.62 (ddd, *J* = 5.0, 1.9, 0.9 Hz, 1H), 7.85–7.77 (m, 2H), 7.57 (d, *J* = 8.0 Hz, 1H), 7.37 (ddt, *J* = 7.0, 4.9, 1.0 Hz, 1H), 6.78 (s, 1H), 6.43 (q, *J* = 7.2 Hz, 1H), 4.31 (t, *J* = 5.6 Hz, 2H), 3.82–3.63 (m, 2H), 3.57–3.47 (m, 5H), 3.46–3.42 (m, 2H), 3.19 (t, *J* = 4.9 Hz, 2H), 2.30 (t, *J* = 6.3 Hz, 2H), 2.28 (s, 3H), 2.15 (d, *J* = 7.2 Hz, 3H), 2.09 (s, 3H). ^13^C NMR (101 MHz, Methanol-*d*_4_) δ 174.14, 168.06, 160.87, 160.59, 156.89, 155.97, 150.62, 141.35, 139.24, 133.29, 132.31, 129.79, 125.55, 124.29, 123.14, 122.76, 117.24, 113.63, 102.38, 70.78, 67.82, 56.57, 51.48, 42.65, 38.94, 37.58, 18.18, 11.45, 10.59. LCMS Method A (Rt = 4.78 min) *m/z* 600.4 [M+1]^+^.

##### (2S,4R)-1-((2S)-2-(3-((1-(2-(3-((2-(7-(3,5-dimethylisoxazol-4-yl)-8-methoxy-2-oxo-1-((R)-1-(pyridin-2-yl)ethyl)-1H-imidazo[4,5-c]quinolin-3(2H)-yl)ethyl)amino)-3-oxopropoxy)ethyl)-1H-1,2,3-triazol-4-yl)methoxy)propanamido)-3,3-dimethylbutanoyl)-4-hydroxy-N-(4-(4-methylthiazol-5-yl)benzyl)pyrrolidine-2-carboxamide (IBET151-VHL-PROTAC):

**Figure:**
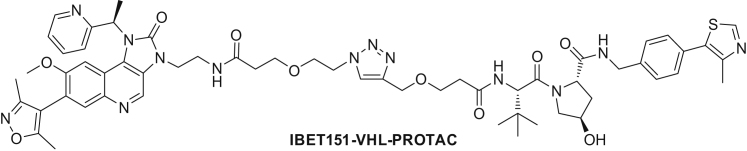

IBET151-Az (50 mM in DMSO, 260 μL, 0.013 mmol) and VHL-alkyne (50 mM in DMSO, 260 μL, 0.013 mmol) were mixed, diluted with *tert*-butanol (260 μL) and then a premixed solution of THPTA (50 mM in water, 104 μL, 5.20 μmol) and copper (II) sulfate (100 mM in water, 26.0 μL, 2.60 μmol) was added. Finally, a freshly prepared solution of sodium *L*-ascorbate (200 mM in water, 65.0 μL, 0.013 mmol) was added. The reaction mixture was stirred at room temperature for 3.5 hr and then it was directly purified by mass directed preparative HPLC. This gave IBET151-VHL-PROTAC formic acid salt (13.6 mg, 88% yield) as a white solid. ^1^H NMR (400 MHz, Chloroform-*d*) δ 8.84 (s, 1H), 8.66 (s, 1H), 8.63 (d, *J* = 4.8 Hz, 1H), 7.83 (s, 1H), 7.81 (s, 1H), 7.66 (t, *J* = 6.0 Hz, 1H), 7.61 (t, *J* = 7.6 Hz, 1H), 7.38–7.29 (m, 5H), 7.23 (dd, *J* = 7.4, 4.9 Hz, 1H), 7.20–7.13 (m, 1H), 7.04 (d, *J* = 8.8 Hz, 1H), 6.71 (s, 1H), 6.44–6.33 (m, 1H), 4.73–4.43 (m, 8H), 4.38 (dd, *J* = 15.1, 5.5 Hz, 1H), 4.34–4.19 (m, 2H), 4.07 (d, *J* = 11.1 Hz, 1H), 3.82–3.45 (m, 12H), 2.50–2.44 (m, 5H), 2.39 (ddd, *J* = 13.1, 8.6, 4.4 Hz, 1H), 2.27 (s, 3H), 2.23–2.06 (m, 9H), 0.95 (s, 9H). ^13^C NMR (101 MHz, Chloroform-*d*) δ 171.87, 171.50, 171.47, 171.18, 166.60, 159.74, 155.74, 150.44, 149.52, 144.54, 138.42, 137.53, 131.01, 130.55, 129.55, 128.11, 124.37, 122.92, 121.57, 112.37, 99.79, 97.13, 70.19, 69.04, 66.90, 66.45, 64.84, 59.01, 57.72, 57.36, 55.89, 50.17, 43.26, 41.88, 38.48, 36.90, 36.72, 35.52, 26.56, 16.20, 11.85, 10.93. LCMS Method A (Rt = 1.83 min) *m/z* 570.9 [M+2]^2+^ (small: 1140.5 [M+1]^+^).

#### Experimental procedures

##### Fluorescence *in situ* hybridization

THP-1 cells have been cultured as described above. Cells were treated with either vehicle, JQ1-Az (10 μM), JQ1-VHL PROTAC (1 μM), JQ1-VHL PROTAC (10 μM) or IBET151-PROTAC (10 μM) for 6 h and afterward RNA fluorescence *in situ* hybridization (FISH) was performed according to a published procedure (Pollex et al., 2013) using a 50bp Cy3-labeled oligo dT probe (Sigma) detecting poly(A)^+^RNA with following variations: 15% formamide was used instead of 50%, and THP-1 cells were immobilized on microscopy slides after fixation using Cytospin (1500 rpm, 5 min, Thermo Fisher). Additionally, staining with Hoechst 33258 (Sigma) and Wheat Germ Agglutinin (WGA) Alexa Fluor 647 Conjugate (Life Technologies) was performed to enable quantification of the data.

Images were acquired with a Zeiss LSM 780 NLO microscope (EMBL, Heidelberg) with excitation at 633, 514 and 405 nm using a 63x oil objective and the auto-focus function. Images were then analyzed using ImageJ (1.45 s) and CellProfiler (2.1.1). In short, a Hoechst staining was used to identify the nucleus and a WGA staining to identify the cell border via the outer cell membrane. The cytosol area was then identified as a difference between cell border and nucleus. The mean fluorescence intensity of the Cy3 probe (514 nm, detecting the poly(A)^+^RNA probe) was then measured at single cell level using the previously defined regions of nucleus and cytosol, and the ratio between both compartments was calculated. On average, 500-700 cells were quantified for each condition.

##### Immunoprecipitation of GREB1

For the enrichment of GREB1 500 μL MCF-7 cell extracts corresponding to 1 mg protein were prepared by lysing the cells in 50 mM Tris–HCl, pH 7.5 (5% (w/v) glycerol, 1.5 mM MgCl2, 150 mM NaCl, 25 mM NaF, 1 mM sodium vanadate detergent (IGEPAL CA-630) at a concentration of

0.8% (w/v), EDTA-free tablet, protease inhibitor cocktail (Roche Diagnostics) per 25 mL of lysis buffer) and incubated for 1 h at 4°C with 10 μM 17-AAG or vehicle (DMSO) followed by incubation with the according antibodies (10 μg anti-GREB1, Abcam, or IgG rabbit control) for two additional hours. Antibodies were captured by incubation with Protein G beads (Sigma) for 1 h and bound proteins were eluted with 80 μL 2x SDS sample buffer and subjected to SDS gel electrophoresis. Samples were further processed for LC-MS/MS analysis.

##### SDS-PAGE and western blot

Jurkat cells treated with 17-AAG (10 μM) or vehicle for 6 h were lysed in SDS-based sample buffer and analyzed by SDS gel electrophoresis. Western blots were probed with antibodies against GLUL (BD Biosciences), HSP70 (Cell Signaling), or α-tubulin (Sigma), and the fluorescence signal of the IRDye secondary antibodies (Odyssey system, Li-Cor Biosciences) was analyzed quantitatively (Odyssey software). Statistics were calculated using a one-sample t test.

##### PCR

mRNA of Jurkat cells treated with 17-AAG (10 μM) or vehicle for 3 or 7 h was purified using the RNAquous extraction kit (Life Technologies), quantified using Qubit kit (Life Technologies), reverse transcribed (Maxima First Strand cDNA kit, Thermo Fisher), and analyzed by qPCR (Step One Real-Time PCR System and software V2.3, Thermo Fisher) using primers targeting GLUL (FAM, Thermo Fisher) and RPL7 (VIC) and the TaqMan master mix (Thermo Fisher) in 96-well reaction plates (Invitrogen). The amplification of GLUL cycle time (C_T_) was normalized to RPL7, yielding ΔC_T_, and the data are displayed as 40-ΔC_T_.

##### Kinobeads assays

Competition binding assays were performed as described previously by using kinobeads (Bantscheff et al., 2007, Werner et al., 2012). Briefly, 50 μL (240 μg protein) cell extract was pre-incubated with test compound or vehicle for 45 min at 4°C followed by incubation with kinobeads (8 μL beads per sample) for 1 hr at 4°C. The beads were washed with lysis buffer and eluted with 20 μL 2 × SDS sample buffer and subjected to SDS gel electrophoresis. Samples were further processed for LC-MS/MS analysis.

##### 2D-TPP experiments, in cells

Experiments were performed as described (Becher et al., 2016). DMSO (vehicle), JQ1 or I-BET151 dissolved in DMSO at final concentrations of 20.0 μM, 5.0 μM, 1.0 μM and 0.1 μM were added to THP1 cells (two plates for each concentration) and incubated for 90 min at 37°C and 5% CO_2_. All following steps were performed as previously described (Becher et al., 2016). The heat treatment was done at 12 temperatures spread across a temperature range of 42.0–63.9°C.

##### 2D-TPP experiments, in cell extracts

Experiments were performed as described (Becher et al., 2016) with slight modifications. THP1 cells were resuspended in PBS. Following resuspension, cells were Dounce homogenized with 20 strokes and incubated with benzonase for 1 h. Protein concentration was determined by Bradford assay (BioRad). 1.5 mL lysate (concentration 2 mg/mL) was distributed to 5 tubes and incubated with vehicle (DMSO) or JQ1 (20.0 μM, 5.0 μM, 1.0 μM and 0.1 μM) for 15 min at 25°C, then 100 μL per well of each reaction was transferred to a 96-well PCR plate. Samples were heated in parallel for 3 min to one of the twelve tested temperatures covering a range of 42.0–60.1°C. Heat-treated samples were then incubated for 3 min at room temperature following addition of IGEPAL CA630 to a concentration of 0.8% and incubated for 1 h with benzonase (4°C at 750 rpm). Aggregated proteins were separated by ultracentrifugation (20 min / 10.000 x g). Samples were further processed for LC-MS/MS analysis.

##### TPP-temperature range (TR) experiments, in cells

Thermal proteome profiling was performed as described (Franken et al., 2015, Savitski et al., 2014). Briefly, HepG2 cells were treated with either vehicle or JQ1 (15 μM), Jurkat cells and T cells were vehicle treated for 90 min at 25°C before heating in parallel for 3 min to the temperature range from 40.0 - 66.3°C. Samples were further processed for LC-MS/MS analysis.

##### TPP-temperature range (TR) experiments, Recombinant proteins

10 nM recombinant FYTTD1 was spiked-in into a background of recombinant protein domains (80 μg/mL final protein concentration) in 20 mM HEPES (pH 7.0), 150 mM NaCl, 5% glycerol and 0.02% Tween20. DMSO (vehicle) or JQ1 (100 μM) was added. Heat treatment using a temperature range from 37°C to 70°C was done as described before (Becher et al., 2016) and aggregates were removed by centrifugation through 100 kD MWCO filter plates. Samples were further processed for LC-MS/MS analysis.

##### Sample preparation for MS, Gel-based

Gel lanes were cut into three slices covering the entire separation range (∼2 cm) and subjected to in-gel tryptic digestion (Bantscheff et al., 2007). Peptides were either directly subjected to LC-MS/MS analysis or labeled via reductive dimethylation or with isobaric mass tags (TMT10, Thermo FisherScientific, Waltham, MA). Recombinant protein samples were digested in solution (2.4 M Urea, 100 mM HEPES pH8, 5 mM CAA, 1.7 mM TCEP, 0.1 μg Trypsin, 0.1 μg LysC).

##### Sample preparation for MS, Isobaric mass tag labeling

TMT labeling was performed using the 10-plex TMT reagents, enabling relative quantification of 10 conditions in a single experiment (Werner et al., 2012, Werner et al., 2014). The labeling reaction was performed in 40 mM triethylammoniumbicarbonate, pH 8.53 at 22°C and quenched with glycine. Labeled peptide extracts were combined to a single sample per experiment.

##### Sample preparation for MS, Dimethyl labeling

Reductive dimethylation was perfomed as described in Boersema et al. (2009). Briefly, peptide digests were resuspended in 30 μL 50 mM TEAB before addition of 5 μL of the corresponding stable isotope labeled fromaldehyde and incubated for 2 min at RT. 5 μL of sodium cyanoborhydride was added following 30 min incubation. Again, 5 μL of sodium cyanoborhydride were added and further incubated. Reactions were quenched by addition of 15 μL 1% ammonia solution before careful acidification by formic acid. According to the schema depicted in Figure 6 human primary CD3+ T cells were isolated from PBMCs and accustomed to SILAC light medium as described above. Then medium was exchanged to heavy SILAC under vehicle (1) or 17-AAG treatment (3), and to SILAC light medium under vehicle treatment (2). Peptides derived from the reference condition maintained in light SILAC medium (2) were labeled by reductive dimethylation using CH_2_O and NaBH_3_CN while vehicle (1) and 17-AAG (3) treated samples subjected to heavy SILAC medium switch were labeled with ^13^CD_2_O and NaBH_3_CN to yield dimethylated N-termini and lysine residues containing (^13^CD_2_H)_2_ (1), (CH_3_)_2_ (2), (^13^CD_2_H)_2_ (3) respectively. Subsequently labeled peptide extracts from (1) was mixed with an aliquot of (2), and (3) was mixed with another aliquot of (2) and both samples were analyzed separately by LC-MS/MS. The analysis of sample (1-2) provided data for new synthesis and degradation of proteins under vehicle treatment, while the analysis of sample (2-3) represented 17-AAG treatment. Presence of vehicle controls (2) in both experiments enabled cross comparison of synthesis and degradation rate changes between vehicle and 17-AAG treatment conditions.

##### Sample preparation for MS, Pre-fractionation

Lyophilized samples were re-suspended in 1.25% ammonia in water. The whole sample was injected onto a pre-colum (2.1 mm x 10 mm, C18, 3.5 μm [Xbridge (Waters, Milford, MA)]) at a flow rate of 15 μL min^-1^. Separation was done at 40 μL min^-1^ on a reversed-phase-column (1 mm x 150 mm, C18, 3.5 μm [Xbridge (Waters, Milford, MA)]) with a gradient of 115 min length ranging from 97% buffer A (1.25% ammonia in water) to 60% B (1.25% ammonia in 70% acetonitrile in water). Fractions were collected for 120 s (9 fractions) or 60 s (25 fractions). After reaching the desired number of fractions (n) the next fraction (n+1) was pooled with the first fraction and so on until the gradient has finished.

##### LC-MS/MS analysis

Samples were dried in vacuo and resuspended in 0.05% trifluoroacetic acid in water. Half of the sample was injected into an Ultimate3000 nanoRLSC (Dionex, Sunnyvale, CA) coupled to a Q Exactive (Thermo Fisher Scientific). Peptides were separated on custom-made 50 cm × 100 μm (ID) reversed-phase columns (Reprosil) at 55°C. Gradient elution was performed from 2% acetonitrile to 40% acetonitrile in 0.1% formic acid and 3.5% DMSO over 2 hr. Samples were online injected into Q-Exactive mass spectrometers operating with a data-dependent top 10 method. MS spectra were acquired by using 70.000 resolution and an ion target of 3E6. Higher energy collisional dissociation (HCD) scans were performed with 35% NCE at 35.000 resolution (at m/z 200), and the ion target settings was set to 2E5 so as to avoid coalescence (Werner et al., 2014). The instruments were operated with Tune 2.2 or 2.3 and Xcalibur 2.7 or 3.0.63.

Synchronous precursor selection (SPS) MS^3^ experiments were performed on an Orbitrap Fusion Lumos operated with fixed cycle time of 2 s. MS spectra were acquired at 60.000 resolution and an ion target of 4E5. Tandem mass spectra were acquired in the linear ion trap following HCD fragmentation performed with 5% stepped collision energy around 38% NCE and an ion target 1E4. MS3 were performed synchronously on the 5 most abundant MS2 signals in the range of 400-2000 m/z using HCD at 70% NCE in the Orbitrap at a resolution of 30.000 and an ion target of 1E5. The instrument was operated with Tune 2.1.1565.23 and Xcalibur 4.0.27.10.

### Quantification and Statistical Analyses

#### Peptide and protein identification

Mascot 2.4 (Matrix Science, Boston, MA) was used for protein identification by using a 10 parts per million mass tolerance for peptide precursors and 20 mD (HCD) mass tolerance for fragment ions. The search database consisted of a customized version of the International Protein Index protein sequence database combined with a decoy version of this database created by using scripts supplied by MatrixScience.

#### Isobaric mass tags

Carbamidomethylation of cysteine residues and TMT modification of lysine residues were set as fixed modifications. Methionine oxidation, and N-terminal acetylation of proteins and TMT modification of peptide N-termini were set as variable modifications.

#### Pulsed SILAC

Carbamidomethylation was set as fixed modification whereas lysine ^13^C_6_^15^N_2_, and arginine ^13^C_6_^15^N_4_, and methionine oxidation and N-terminal acetylation of proteins were set as variable modifications.

#### Multiplexed pulsed SILAC isobaric mass tags

Searches for light and heavy SILAC were perfomed independently. Carbamidomethylation of cysteine residues and TMT modification of lysine residues were set as fixed modifications for the SILAC light search. Carbamidomethylation, lysine ^13^C_6_^15^N_2_ with TMT, and arginine ^13^C_6_^15^N_4_ were set as fixed modifications in the heavy SILAC search. Methionine oxidation, N-terminal acetylation of proteins and TMT modification of peptide N-termini were set as variable modifications in both searches.

#### Multiplexed pulsed SILAC Dimethyl

Searches were perfomed with Carbamidomethylation as fixed modifications and Dimethyl (CH_3_)_2_, Lysine ^13^C_6_^15^N_2_ with dimethyl (CH_3_)_2_, lysine ^13^C_6_^15^N_2_ with dimethyl (^13^CHD_2_)_2_, arginine ^13^C_6_^15^N_4_, methionine oxidation and N-terminal acetylation of proteins as variable modifications.

Unless stated otherwise, we accepted protein identifications as follows: (i) For single-spectrum to sequence assignments, we required this assignment to be the best match and a minimum Mascot score of 31 and a 10 × difference of this assignment over the next best assignment. Based on these criteria, the decoy search results indicated < 1% false discovery rate (FDR). (ii) For multiple spectrum to sequence assignments and using the same parameters, the decoy search results indicated < 0.1% FDR. Quantified proteins were required to contain at least 2 unique peptide matches. FDR for quantified proteins was < 0.1%.

#### Peptide and protein quantification

##### Isobaric mass tag based quantification

Reporter ion intensities were read from raw data and multiplied with ion accumulation times (the unit is milliseconds) so as to yield a measure proportional to the number of ions; this measure is referred to as ion area (Savitski et al., 2013). Spectra matching to peptides were filtered according to the following criteria: mascot ion score > 15, signal-to-background of the precursor ion > 4, and signal-to-interference > 0.5 (Savitski et al., 2013). Fold changes were corrected for isotope purity as described and adjusted for interference caused by co-eluting nearly isobaric peaks as estimated by the signal-to-interference measure (Savitski et al., 2013). Protein quantification was derived from individual spectra matching to distinct peptides by using a sum-based bootstrap algorithm; 95% confidence intervals were calculated for all protein fold changes that were quantified with more than three spectra (Savitski et al., 2013).

##### Peptide precursor intensity based quantification

All acquired raw data were processed using a modified version of isobarQuant available from the Github code repository https://github.com/protcode/isob/archive/1.1.0.zip: further details on the processing will be published separately. Unique quantified peptides associated with each protein group were selected and filtered according to the settings given in the isobarQuant configuration file (proteinquantfication.cfg): Mascot score > 15, peptide length ≥ 6, FDR at Mascot score < 1%, maximum least-squares fit of both peptides in light/heavy pair ≤ 0.1 and maximum prior ion ratio of both peptides in light/heavy pair ≤ 0.2. Protein fold changes were calculated using the median of all valid quantified peptide ratios (the fitted intensity of heavy SILAC to light SILAC) linked to the protein group. For protein groups where there was a minimum of one peptide with a positive ratio, any peptides with an undetermined ratio were excluded from the median calculation. In some cases, due to the slow protein turnover in T cells, proteins that were robustly quantified in the vehicle condition were not identified in the 17-AAG condition due to strong downregulation. In such cases we used half of the smallest detected relative protein abundance across the experiment as a proxy for the abundance of the non-identified proteins in order to be able to calculate the fold change and to capture that the protein was downregulated by 17-AAG. Proteins were filtered out in case the effect between the 2 donors in either vehicle control or 17-AAG differed by more than 8-fold or if the quantification was based on less than 3 quantified spectrum sequence matches. Changes in nascent and mature protein concentrations were calculated in the vehicle and 17-AAG experiments and compared to each other in order to determine 17-AAG induced synthesis and degradation changes. Experiments were repeated for 2 independent donors in order to rule out donor to donor variations.

#### Analysis of TPP TR and 2D-TPP experiments

Experiments were analyzed as described in Becher et al. (2016) and Franken et al. (2015).

##### TPP-TR

Following LC-MS/MS analysis, protein fold changes were computed relative to the protein abundance at the lowest temperature. These fold changes represent the relative amount of nondenatured protein at the corresponding temperature. After a global normalization procedure, melting curves were fitted to the fold changes of each individual protein according to the formula

$$f\left(T\right)=\frac{1-plateau}{1+e^{-\left(\frac{a}{T}-b\right)}}+plateau$$

where T is the temperature and a, b, and plateau are constants. Several parameters were derived that are relevant for the subsequent significance analysis:

• The coefficient of determination R^2^, which indicates how well the fold changes fit the melting curve.
• The melting point (T_m_) of the protein under the corresponding condition, which is given by the temperature at which the value of the melting curve is 0.5.
• The slope of the curve at its steepest point (i.e., the inflection point).
• The plateau of the fitted melting curve, which is given by its lower horizontal asymptote.

A change in the thermal stability of a protein is indicated by a compound-induced shift of its melting curve, which becomes apparent in a difference between the melting points derived under vehicle- and compound-treated conditions.

For a protein to be considered in the statistical evaluation, we required the R^2^ of both the vehicle- and the compound-treated melting curves to be > 0.8 and the plateau of the vehicle curve to be < 0.3 in our analysis. The slope of the melting curve has a substantial impact on the reproducibility of the melting point difference. Thus, the slope has a central role in our data quality–dependent significance assessment. We divided the considered set of proteins into bins, starting with the shallowest slope, so that each bin contained at least 300 proteins. Then, we used z-tests to assess the statistical significance of melting point differences for the proteins one bin at a time, before performing the Benjamini-Hochberg correction for multiple testing on the full dataset. For a compound-induced change in a protein’s thermal stability to be regarded as significant, it was required to fulfill the following criteria:

• One of the p values for the two replicate experiments is < 0.05 and the other one is < 0.1.
• The melting point shifts in the two vehicles versus treatment experiments have the same direction (i.e., the protein was either stabilized or destabilized in both cases)
• Both melting point differences in the two pairs of control versus treatment experiments are greater than the melting point difference between the two vehicle controls.
• The minimum slope in each of the control versus treatment experiments is < −0.06.

##### 2D-TPP

For each of the experiments that underwent heat treatment at one of the 12 different temperatures (see above) we performed the following analysis: Following LC-MS/MS analysis, fold changes were computed to the protein abundance at the lowest compound concentration (vehicle). These fold changes represent the protein’s apparent stability at the corresponding compound concentration. If a protein’s fold change at the highest compound concentration was > 3/2, we regarded the protein to be potentially stabilized by the compound. Conversely, if a protein’s fold change at the highest compound concentration was < 2/3, we regarded the protein to be potentially destabilized by the compound. Otherwise, we did not consider the protein for further analysis. Subsequently, the fold changes were transformed such that with increasing compound concentration they range from 0 to 1 for stabilized proteins and from 1 to 0 for destabilized proteins. A sigmoidal curve according to the formula

$$Y=\frac{1}{1+10^{\left(\text{log}\;EC_{50}-x\right)\cdot slope}}$$

was fitted.

If the R^2^ of the curve fit was > 0.8, we regarded the observed stabilization or destabilization to be a compound-induced effect as opposed to random fluctuations. In that case, we derived the pEC_50_ as the compound concentration at which the value of the fitted curve is 0.5. If the derived value was below the lowest (nonvehicle) compound concentration (i.e., pEC_50_ < log10(minimum applied compound concentration)), this was reported in the output and that pEC_50_ was not considered valid.

#### Protein half-life determination

Protein half-lives were determined according to a modified version of the protein decay rate method described by Schwanhäusser et al. (2009). The rate constant of the protein decay *k*_*dp*_ was computed according to

$$k_{dp}=\frac{\sum_{i=1}^{m}{\text{log}}_{e}\left(r_{t_{i}}+1\right)\cdot t_{i}}{\sum_{i=1}^{m}t_{i}^{2}}$$

where *m* is the number of time points (*t*_*i*_) considered and *r*_*ti*_ was the fold change ratio (nascent proteins / mature proteins) of a specific protein at each time point. If required *k*_*dp*_ was corrected for the cell cycle time *t*_*cc*_ by extending the formula to

$$k_{dp}=\frac{\sum_{i=1}^{m}{\text{log}}_{e}\left(r_{t_{i}}+1\right)\cdot t_{i}}{\sum_{i=1}^{m}t_{i}^{2}}-\frac{\log_{e}2}{t_{cc}}$$

as described in Schwanhäusser et al. (2009). The half-life (*T*_1/2_) of a protein is then calculated by

$$T_{1/2}=\frac{{\text{log}}_{e}2}{k_{dp}}$$

For each protein a linear model was fitted to the time course of the logarithmic protein fold changes [log_*e*_(*r*_*ti*_+1)] and the coefficient of determination (*R*^2^) for the linear regression was recorded. The QC value was set to ‘weak’ if it was possible to determine a fold change in at least three out of the four time points, ‘good’ if the protein fold changes at three out of the four time points were based on a minimum of three quantified peptides and ‘poor’ for the remainder.

#### Statistical analysis

All experiments have been performed in biological duplicates if not stated otherwise.

##### Statistical analysis of mPDP experiments

Relative fold changes were calculated for all quantified proteins and after log_2_ transformation median normalization was applied to ensure that the data was centered around zero. For cases where a substantial number of the protein population was regulated, as was the case for the nascent proteins upon JQ1-VHL PROTAC treatment, normalization factors calculated for the mature proteins were applied to the nascent proteins. After the normalization proteins were divided into bins according to the number of quantified spectrum sequence matches. Each bin consisted of at least 300 proteins. This data quality–dependent binning strategy is analogous to the procedure described previously (Cox and Mann, 2008). The standard deviation per bin was calculated using robust estimation (using the 15.87, 50, and 84.13 percentiles) from the distribution of differences between the log_2_-transformed fold changes (FC) of the same proteins from two biological replicates divided by the square root of two:

$$\frac{\left({\text{log}}_{2}\left({\text{FC}}_{\text{rep}1}\right)-{\text{log}}_{2}\left({\text{FC}}_{\text{rep}2}\right)\right)}{\sqrt{2}}$$

The metric measures the shortest distance to the equality line. By using the standard deviation from the differences in log_2_ fold changes of proteins between two biological replicates the reproducibility between the replicates is taken into account in the statistical test. This enables to assess biologically relevant regulation upon perturbation without having to adhere to the assumption that the majority of measured proteins are not regulated. The basis for the p value calculations is the same as for the MaxQuant (Cox and Mann, 2008) significance estimation. For each protein log_2_ fold change a p value was calculated using a Z-test with a robust estimation of the standard deviation calculated as described above. The background data used for p value estimation had symmetrical bell shape distributions in all cases. No additional methods for assessing normality were applied. When p values have been calculated for all bins, adjustment for multiple hypothesis testing was performed on the full dataset by using Benjamini-Hochberg correction. Proteins were counted as significantly regulated if the adjusted p value was ≤ 0.05 and if the protein expression in both replicates changed by at least by 30% in the same direction. Statistical analysis and visualization of the data were performed using the statistical language R, Tibco Spotfire 7.0.1.24 and Graphpad Prism 7.

#### Boxplots

Boxplots shown were generated using the statistical language R and the ggplot2 package. The lower and the upper hinges of the boxes correspond to the 25% and the 75% percentile, the bar in the box shows the median. The upper whisker extends to the largest value but maximal 1.5 times the IQR from the upper hinge. Similarly, the lower whisker extends from the lower hinge at most 1.5 IQR. Points outside of the whiskers are plotted individually as outliers.

Group tests were performed using a nonparametric Wilcoxon test and statistical significance is indicated by: ^∗^ p ≤ 0.05, ^∗∗^ p ≤ 0.01 and ^∗∗∗^ p ≤ 0.001 if not stated otherwise.

##### Clustering

Clustering of proteins significantly regulated when comparing effect upon treatment with estradiol, GW7604 and raloxifene was done using Euclidian distance and complete linkage.

K-means clustering of T cell stimulation expression proteomics was performed with k = 4 clusters. Clustering analysis were done using Tibco Spotfire 7.0.1.24

##### Classification of HSP90 dependence

A protein was considered as HSP90 dependent if it was significantly downregulated (adjusted p value ≤ 0.05 and the protein expression in both replicates reduced by at least by 30% compared to control) in at least one time point. When a protein was significantly downregulated in its mature state at any time point it was classified as constitutively HSP90 dependent irrespective of the effect on nascent protein. Significant downregulation at only nascent state classified the protein as HSP90 dependent during synthesis.

Fisher’s exact test was used to evaluate the significance of enrichment of annotated HSP90 interactors among proteins dependent on constitutively or during synthesis compared to all other identified proteins.

### Data and Software Availability

Mass spectrometry raw data are available via PRIDE using the following accession numbers for the individual datasets Multiplexed proteome dynamics profiling of JQ1-PROTAC: PRIDE: PXD008637, Thermal proteome profiling of JQ1: PRIDE: PXD008638, 2 Dimensional Thermal proteome profiling of JQ1: PRIDE: PXD008626, Multiplexed proteome dynamics profiling of Estradiol and SERMs: PRIDE: PXD008636, Expression proteomics combination treatment of Estradiol and SERMs: PRIDE: PXD008628, Multiplexed proteome dynamics profiling of HSP90 inhibitor 17-AAG: PRIDE: PXD008633, Immunoaffinity enrichment of GREB1 to co-enrich HSP90: PRIDE: PXD008631, Thermal proteome profiling of Jurkat cells: PRIDE: PXD008639, Kinobeads affinity enrichment from lysates derived from stimulated cells: PRIDE: PXD008632, Jurkat protein half live: PRIDE: PXD008629, T cell protein half live: PRIDE: PXD008630, Multiplexed proteome dynamics profiling of HSP90 inhibitor 17-AAG in T cells: PRIDE: PXD008635, Multiplexed proteome dynamics profiling of HSP90 inhibitor 17-AAG in anti-CD3 and anti-CD28 stimulated T cells: PRIDE: PXD008634 and Expression profiling of HSP90 inhibitor 17-AAG in anti-CD3 and anti-CD28 stimulated T cells: PRIDE: PXD008627.

Full datasets for individual figures containing e.g., fold changes and p values were uploaded to Mendeley Data and can be retrieved using the following link https://doi.org/10.17632/8pzhg2tdyb.1.

## Supplemental Information

Table S1. GREB1 Immunoprecipitation, Related to Figure 4

Table S2. Proteostasis Effects of Compound Treatment on Proteins Encoded by Estrogen-Responsive Genes, Related to Figure 4

Table S3. Protein List and HSP90 Dependence, Related to Figures 5, 6, and 7

Table S4. Picard List, 2015 Download, and Match to Protein Names in Current Publication, Related to Figures 5, 6, and 7

Table S5. Comparison between HSP90 Co-purifying Proteins and HSP90 Requirement Determination in Respective Cell Types, Related to Figures 5 and S5

Table S6. Nonparametric Wilcoxon-Test-Derived p Values for Protein Groups and Group Size, Related to Figures 5 and 6
