# Supplementary material for: Multiplexed Proteome Dynamics Profiling Reveals Mechanisms Controlling Protein Homeostasis
Source: Cell. 2018 Mar 22;173(1):260–274.e25. doi: 10.1016/j.cell.2018.02.030 (PMC5871718; doi:10.1016/j.cell.2018.02.030)
Supplement: Table S2. Proteostasis Effects of Compound Treatment on Proteins Encoded by Estrogen-Responsive Genes, Related to Figure 4 [file mmc2.pdf]

Table S2, Related to Figure 4

Proteostasis effects of compound treatment on proteins encoded by Estrogen responsive genes

A) Significant changes compared to vehicle control

(↑ increased, ↔ no change, ↓ decreased)

Protein	Literature	Maturation state	Estradiol	Raloxifene	GW5638	17-AAG
GREB1	(Charpentier et al., 2000; Laviolette et al., 2014)	Mature	↑	↓	↓	↓
		Nascent	↑	↓	↓	↓
CAV1	(Charpentier et al., 2000; He et al., 2016)	Mature	↔	↔	↔	↑
		Nascent	↑	↓	↓	↔
CCNB1	(Charpentier et al., 2000; O'Donnell et al.)	Mature	↔	↔	↔	↔
		Nascent	↔	↔	↔	↑
SLC7A5	(Charpentier et al., 2000)	Mature	↔	↔	↔	↔
		Nascent	↑	↔	↔	↑
FOSL2	(Lin et al., 2007; Stender et al., 2010)	Mature	↔	↔	↔	↔
		Nascent	↑	↔	↓	↔
ERBB2	(Wärri et al., 1991)	Mature	↔	↔	↔	↓
		Nascent	↓	↔	↔	↓

B) Significant changes compared to Estradiol (↑ increased, ↔ no change, ↓ decreased)

Protein	Maturation state	Raloxifene	GW5638
GREB1	Mature	↓	↓
	Nascent	↓	↓
CAV1	Mature	↔	↔
	Nascent	↓	↓
CCNB1	Mature	↔	↔
	Nascent	↓	↓
SLC7A5	Mature	↔	↔
	Nascent	↓	↓
FOSL2	Mature	↔	↔
	Nascent	↓	↓
ERBB2	Mature	↔	↔
	Nascent	↑	↑

Charpentier, A.H., Bednarek, A.K., Daniel, R.L., Hawkins, K.A., Laflin, K.J., Gaddis, S., MacLeod, M.C., and Aldaz, C.M. (2000). Effects of Estrogen on Global Gene Expression: Identification of Novel Targets of Estrogen Action. Cancer Res. *60*, 5977–5983.

He, L., Hu, X.-T., Lai, Y.-J., Long, Y., Liu, L., Zhu, B., and Chen, G.-J. (2016). Regulation and the Mechanism of Estrogen on Cav1.2 Gene in Rat-Cultured Cortical Astrocytes. J. Mol. Neurosci. *60*, 205–213.

Laviolette, L.A., Hodgkinson, K.M., Minhas, N., Perez-Iratxeta, C., and Vanderhyden, B.C. (2014). 17β-estradiol upregulates GREB1 and accelerates ovarian tumor progression in vivo. Int. J. Cancer *135*, 1072–1084.

Lin, C.-Y., Vega, V.B., Thomsen, J.S., Zhang, T., Kong, S.L., Xie, M., Chiu, K.P., Lipovich, L., Barnett, D.H., Stossi, F., et al. (2007). Whole-Genome Cartography of Estrogen Receptor α Binding Sites. PLOS Genet. *3*, e87.

O'Donnell, A.J.M., Macleod, K.G., Burns, D.J., Smyth, J.F., and Langdon, S.P. Estrogen receptor-α mediates gene expression changes and growth response in ovarian cancer cells exposed to estrogen.

Stender, J.D., Kim, K., Charn, T.H., Komm, B., Chang, K.C.N., Kraus, W.L., Benner, C., Glass, C.K., and Katzenellenbogen, B.S. (2010). Genome-Wide Analysis of Estrogen Receptor α DNA Binding and Tethering Mechanisms Identifies Runx1 as a Novel Tethering Factor in Receptor-Mediated Transcriptional Activation. Mol. Cell. Biol. *30*, 3943–3955.

Wärri, A.M., Laine, A.M., Majasuo, K.E., Alitalo, K.K., and Härkönen, P.L. (1991). Estrogen suppression of erbB2 expression is associated with increased growth rate of ZR-75-1 human breast cancer cells in vitro and in nude mice. Int. J. Cancer *49*, 616–623.
