# Supplementary material for: Multiplexed Proteome Dynamics Profiling Reveals Mechanisms Controlling Protein Homeostasis
Source: Cell. 2018 Mar 22;173(1):260–274.e25. doi: 10.1016/j.cell.2018.02.030 (PMC5871718; doi:10.1016/j.cell.2018.02.030)
Supplement: Table S5. Comparison between HSP90 Co-purifying Proteins and HSP90 Requirement Determination in Respective Cell Types, Related to Figures 5 and S5 [file mmc5.pdf]

Table S5, Related to Figures 5, S5

Comparison between HSP90 co-purifying proteins and HSP90 requirement determination in respective cell types. Related to Figure 5, S5

Cells	HSP90 co-purifying	Clients			
		Constitutive	Synthesis	No	Total
Jurkat	Yes	52<br>$p < 2.2e-16$	95<br>$p = 4.7e-15$	168	315
	No	137	812	4350	5299
	Total	189	907	4518	5614

MCF-7	Yes	72<br>$p < 2.2e-16$	69<br>$p = 4e-13$	206	347
	No	299	590	5571	6460
	Total	371	659	5777	6807

P-values are derived using Fisher's exact test comparing clients to non-clients.
